# Cortical Region Reporting Patterns in Neurodevelopmental Disorders: A Systematic Review of fNIRS Studies

**DOI:** 10.3390/bios16080408

**Published:** 2026-07-27

**Authors:** Umm E. Habiba, Nida Mateen, Keum-Shik Hong, Chang-Seok Kim, Jing Meng, Hwidon Lee, Jeesu Kim

**Affiliations:** 1 Department of Cogno-Mechatronics Engineering, Pusan National University, Busan 46241, Republic of Korea; uhabiba@pusan.ac.kr (U.E.H.); nidamateen@pusan.ac.kr (N.M.); ckim@pusan.ac.kr (C.-S.K.); 2 Institute for Future, School of Automation, Qingdao University, Qingdao 266071, China; kshong@pusan.ac.kr; 3 Institute for Industrial Artificial Intelligence, Pusan National University, Busan 46241, Republic of Korea; 4 Engineering Research Center for Color-Modulated Extra-Sensory Perception Technology, Crystal Bank Research Institute, Pusan National University, Busan 46241, Republic of Korea; 5 School of Computer Science, Qufu Normal University, Rizhao 276826, China; jingmeng@qfnu.edu.cn

**Keywords:** functional near-infrared spectroscopy (fNIRS), neurodevelopmental disorders, prefrontal cortex, neuroimaging

## Abstract

Functional near-infrared spectroscopy (fNIRS) is a portable, non-invasive tool for studying cortical function in children with neurodevelopmental and neurological disorders. Although fNIRS use is increasing, heterogeneous study paradigms and cortical targets have limited cross-condition comparisons and the identification of shared research priorities. This systematic review maps cortical regions and reporting patterns in five key conditions—autism spectrum disorder (ASD), attention-deficit/hyperactivity disorder (ADHD), cerebral palsy (CP), hypoxic–ischemic encephalopathy (HIE), and epilepsy (Ep)—from January 2015 to December 2025. A systematic search across five databases identified 72 relevant studies meeting PRISMA 2020 criteria, revealing both similarities and differences across disorders. The prefrontal cortex (PFC) was studied in all five conditions (5/5: 100%), making it the most consistently investigated cortical region. The parietal and temporal cortices were studied in 4 of 5 conditions (80%). The frontal cortex had the most regions investigated, while the temporal cortex showed the most consistent coverage across conditions (2.25 conditions per region). Beyond regional preferences, condition-specific patterns were aligned with their disorder phenotypes: social brain networks in ASD, prefrontal executive systems in ADHD, sensorimotor changes in CP, cerebrovascular monitoring in HIE, and state-dependent changes in Ep. Across conditions, researchers found altered prefrontal activity, disrupted connectivity, and compensatory brain responses, supporting broader frameworks. Collectively, these findings identify the PFC as a shared target for transdiagnostic fNIRS investigations while highlighting important gaps in regional coverage and methodological consistency. Despite methodological differences, fNIRS shows promise for identifying both shared and unique brain patterns in pediatric neurodevelopmental and neurological disorders. This review provides a framework for prioritizing cortical targets and guiding future standardized fNIRS research in pediatric populations.

## 1. Introduction

Neurodevelopmental disorders represent a group of conditions characterized by early disruptions in brain development, resulting in persistent cognitive, motor, and behavioral impairments. According to symptom patterns and behavior characteristics, conditions such as autism spectrum disorder (ASD), attention-deficit/hyperactivity disorder (ADHD), cerebral palsy (CP), hypoxic–ischemic encephalopathy (HIE), and epilepsy (Ep) are generally defined as neurodevelopmental disorders. Nonetheless, recent research indicates that these diagnostic conditions consistently overlap at the neural level, which directly opposes fixed categorical frameworks of neurodevelopmental pathology [1].

Relying on symptom-based classification systems may limit the adaptation of dimensional neural features that can pose a significant challenge for traditional diagnostic frameworks, thereby obscuring shared neural systems and neurobiological processes across disorders. Recent neurophysiological approaches reveal that connectivity-based analyses (network connectivity mapping) and non-linear neurophysiological dynamics can yield supplementary insights into brain network organization. This also extends the scope beyond conventional symptom-based diagnostic classifications, thereby supporting a more informed biological understanding of neurodevelopmental and neurological disorders [2,3]. As a result, transdiagnostic and dimensional solutions have become increasingly promising tools for modern neuroscience, aiming to identify shared neural substrates that extend beyond diagnostic limitations [4,5]. Within these parameters, identifying transdiagnostic neural biomarkers and neurophysiological signatures that span multiple conditions has become a central goal for improving early detection, risk assessment, and individualized treatment strategies [6,7]. Functional near-infrared spectroscopy (fNIRS) has emerged as a promising technique to examine cortical function in pediatric populations and patients with neurodevelopmental conditions. fNIRS provides a non-invasive indirect measure of cortical hemodynamic activity that can be connected to neurovascular coupling by tracking changes in oxygenated hemoglobin (HbO), deoxygenated hemoglobin (HbR), and total hemoglobin (HbT) during both rest and task-oriented states [8]. fNIRS has also been found very useful when examining populations where conventional neuroimaging methods, such as functional magnetic resonance imaging (fMRI), can be difficult or poorly tolerated, given its motion tolerance, portability, and appropriateness for use in infants and children [9].

Over the past 10 years, the use of fNIRS has expanded from central activation mapping to the study of distributed cortical networks, system-level organization, and functional connectivity, driven by improvements in analysis and instrumentation [8]. Consequently, fNIRS has become increasingly popular and is seen as a system-level neuroimaging approach that can track large-scale cortical hemodynamics in neurodevelopmental disorders. Although there is an expanding amount of fNIRS literature, many of these studies still only focus on specific diagnostic categories and are published individually, with notable changes in experimental tasks, cortical regions of interest, and analytical techniques. This heterogeneity limits the ability to create logical conclusions about shared versus condition-specific cortical targets across neurodevelopmental disorders, and no cross-condition systemic synthesis of regional reporting patterns has been performed. Additionally, some reviews have examined convergent cortical systems and potential transdiagnostic biomarkers by systematically analyzing fNIRS results across multiple neurodevelopmental conditions [10,11]. In this systematic review, we synthesize fNIRS data from ASD, ADHD, CP, and HIE to describe patterns of cortical involvement at the system level. Moreover, Ep can serve as a pathophysiological comparator due to its well-defined cortical dynamics, neurovascular coupling, and hemodynamic responses. It also provides an informative framework to interpret fNIRS-derived results of brain dysfunction [12,13]. Importantly, in this review, Ep is not treated as a neurodevelopmental disorder but rather as a reference condition that provides a pathophysiological understanding of cortical reorganization, lateralization, and network-level changes, which can aid in interpreting findings in developmental disorders.

This systematic review has two aims: (1) to characterize which cortical regions were examined across fNIRS studies of five pediatric neurodevelopmental and neurological conditions (ASD, ADHD, CP, HIE, Ep) from 2015–2025, identifying universally reported regions and condition-specific research priorities; and (2) to synthesize condition-specific cortical targeting patterns to characterize research convergence and disease-specific regional foci. Unlike previous reviews that summarized fNIRS findings within individual disorders [11,14], this review provides the first systematic comparison of cortical reporting patterns across multiple pediatric neurodevelopmental and neurological conditions. To the best of our knowledge, this is the first systematic synthesis of cross-condition cortical reporting patterns in pediatric fNIRS literature. By highlighting both convergent and divergent regional targeting patterns, this systematic review offers a research framework for future researchers to elucidate ideal cortical targets and advance transdiagnostic understanding of neurodevelopmental brain function.

Following this introduction, this review is organized as follows. Section 2 describes the complete methodology of the systematic review. Section 3 presents the evidence synthesis, including common cortical region reporting patterns, disorder-specific cortical profiles, and a comparative synthesis of these reporting patterns. Section 4 discusses the key concepts explored in this review with biological and methodological implications. Section 5 concludes the review by summarizing the key findings, limitations, and future research directions.

## 2. Materials and Methods

### 2.1. Search Strategy

This review was conducted in accordance with the Preferred Reporting Items for Systematic Reviews and Meta-Analyses (PRISMA) 2020 guidelines to ensure methodological transparency and reproducibility [15]. Its goal is to synthesize fNIRS findings across neurodevelopmental and neurological disorders utilizing a harmonized, qualitative, and system-level approach. The study selection process is outlined in a PRISMA 2020 flow diagram [15].

A thorough literature search was conducted across key electronic databases, including Web of Science, PubMed, MEDLINE, Scopus, and Embase, and included studies published from January 2015 to December 2025 (Note S1). This literature search follows established reporting practices where systematic reviews conclude research trends, methodological characteristics, and constraints within emerging fields [16]. Keywords related to fNIRS (such as “fNIRS” and “near-infrared spectroscopy”) were combined with diagnostic terms for developmental and neurological disorders, which entail ASD, ADHD, CP, HIE, and Ep. Conceptual blocks were merged utilizing Boolean operators, and when needed, database-specific subject headings were applied. The Supplementary Search Criteria contains precise search strings and complete search strategies.

### 2.2. Eligibility Criteria

Studies were considered eligible only if they met the following criteria (Note S2): (1) use of fNIRS as the main neuroimaging technique; (2) recruited human subjects who have a confirmed diagnosis of ASD, ADHD, CP, HIE, or Ep; (3) investigated cortical activation and/or functional connectivity outcomes taken from task-based paradigms or resting-state. Both adult and pediatric studies, including observational, descriptive, clinical, randomized controlled, non-randomized, and pilot studies, were considered. However, adult Ep studies were combined only for comparative purposes and were not used to infer interpretations of neurodevelopmental trajectories. If studies specifically focused on brainstem structures or subcortical regions, used animal or simulation models, contained review articles or case studies, or lacked extractable cortical region information, they were excluded from the study. Articles published in English were inspected and added to this review.

### 2.3. Study Selection

**Figure 1:**
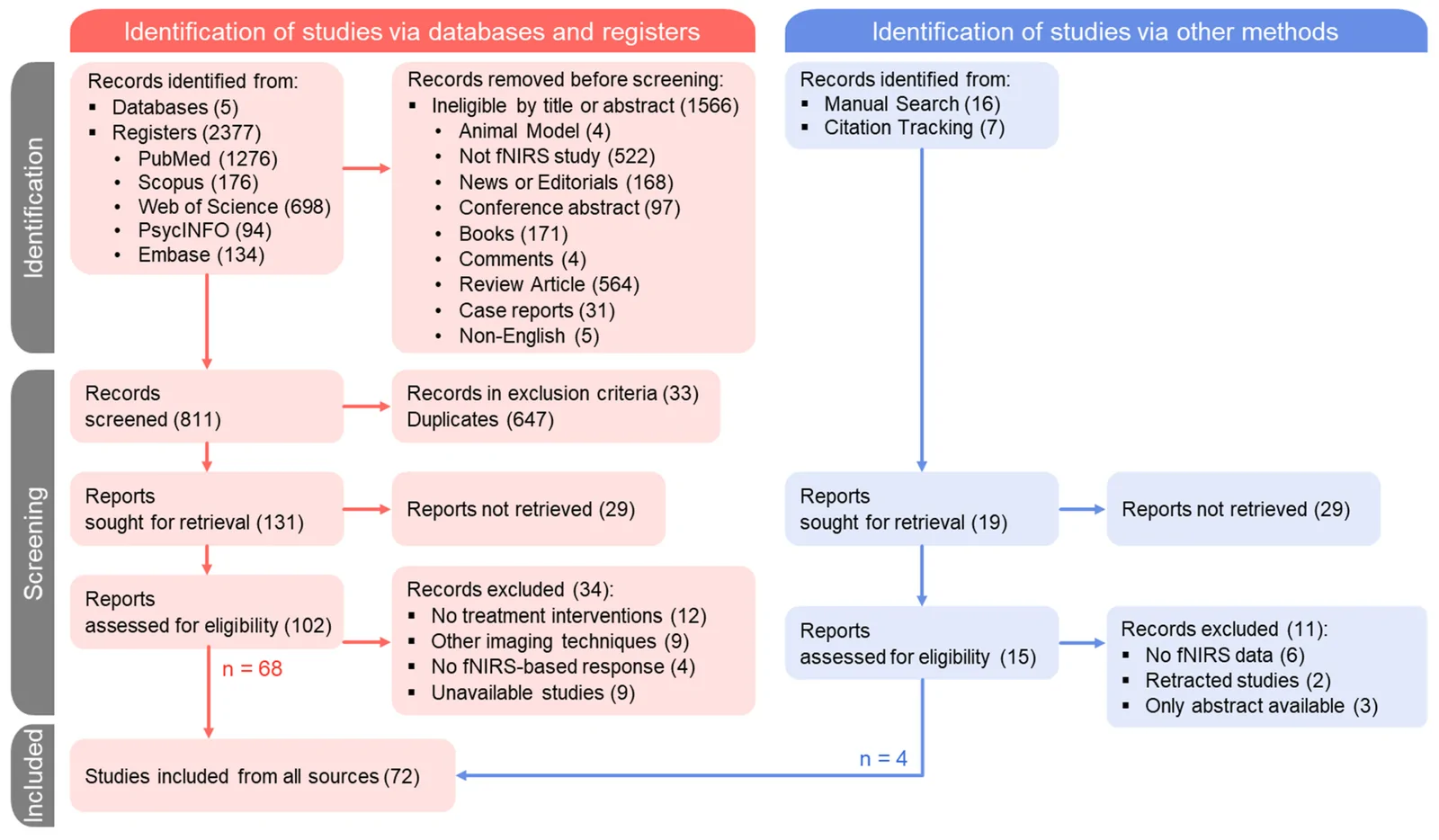
Systematic review process based on the PRISMA 2020 flow diagram for the identification, screening, and selection of eligible studies. Numbers in parentheses are the number of records, reports, or studies. fNIRS, functional near-infrared spectroscopy.

Two independent study reviewers (JK and UEH) conducted database searches, and all reports were transferred into the reference management system, i.e., Rayyan (https://www.rayyan.ai, accessed on 5 December 2025) [17]. Primarily, duplicates were removed before extensive screening. Afterwards, the two reviewers mentioned above conducted title and abstract screening using Rayyan to classify eligible studies for full-text review. Records were categorized as included, excluded, or unclear. Articles that met the initial criteria were rigorously evaluated against the predefined inclusion and exclusion criteria. Certain studies that did not meet the inclusion criteria were excluded from this stage. During the screening process, all discrepancies and confusions were resolved through discussions and consensus. The number of records identified, screened, included, and excluded at every stage is detailed in the PRISMA 2020 flow diagram (Figure 1).

### 2.4. Data Extraction Framework

Systematic data extraction was conducted for each included study using a predefined extraction system. Extracted variables included basic publication information (author, year, country), study design, fNIRS system characteristics (instrument type, number of channels, wavelengths), participant demographics (sample size, age range or age group, and diagnostic category), experimental paradigm (resting-state or task-based), examined cortical regions, and neurophysiological measures (HbO, HbR, HbT, and functional connectivity metrics), as well as the direction of reported effects and hemispheric lateralization. Studies involving Ep were analyzed using the same framework but were analyzed differently, as a pathophysiological comparator rather than being incorporated into the neurodevelopmental disorder group.

### 2.5. Data Synthesis and Harmonization

Due to considerable heterogeneity across studies, including participants’ ages, task paradigms, fNIRS specifications, and analytical approaches, a quantitative meta-analysis was not conducted. Alternatively, a specific qualitative and descriptive synthesis was conducted. Reported cortical regions were organized into anatomical and functional system-level categories to enable cross-study comparisons and mapping. These system-level categories included frontal (prefrontal), parietal, temporal, occipital, sensorimotor, and frontoparietal networks. This abstraction was intended to emphasize the spatial resolution and reporting practices of fNIRS studies rather than precise gyral localization. Qualitative descriptors such as ‘most frequent’ or ‘commonly reported’ were used to showcase recurrence across studies and do not imply quantitative weighting, statistical aggregation, or meta-analytic inference.

### 2.6. Visualization and Transdiagnostic Framework

System-level reporting frequency table and lateral brain-surface schematics were created to envision the cortical regions examined across multiple disorders. These visualizations reflect the frequency with which certain specific cortical systems were studied across conditions and were intended to underscore transdiagnostic convergence, not to show the magnitude of neurophysiological effects. In these visuals, Ep was included only to provide a comparative framework regarding the cortical network and hemispheric reorganization and was not presented as a neurodevelopmental condition. This synthesis was organized within a transdiagnostic framework, highlighting cortical regions repeatedly examined across multiple disorders as potential transdiagnostic neural targets, while also preserving disorder-specific reporting patterns and distinctions. Data was organized into standardized tables (Table 1, Table 2, Table 3, Table 4 and Table 5) and visualized using Python (version 3.12) with Matplotlib (version 3.10). The detailed characteristics of the studies can be found in the disorder-specific section below.

## 3. Results

### 3.1. Search Results and Study Selection

The systematic literature search identified 2377 records across five electronic databases, with an additional 23 records identified through manual searching and citation tracking. After duplicate records were removed, titles and abstracts were screened for relevance. Following full-text assessment based on predefined inclusion and exclusion criteria, 72 studies met eligibility requirements and were included in the final synthesis. The complete study selection process, including reasons for exclusion at each stage, is presented in the PRISMA 2020 flow diagram (Figure 1).

### 3.2. Characteristics of Included Studies

#### 3.2.1. Distribution by Disorder

The final dataset consisted of 72 studies spanning five neurodevelopmental disorders: ASD (*n* = 28; 38.9%), ADHD (*n* = 17; 23.6%), HIE (*n* = 13; 18.1%), CP (*n* = 7; 9.7%), and Ep (*n* = 7; 9.7%). Out of all the disorders, ASD emerged as the most extensively studied, representing over one-third of the literature. This reflects sustained research interest in understanding deficits in social cognition, language processing, and executive functioning using fNIRS methods. Additionally, ADHD was the second most focused group, with studies largely examining attention networks, inhibitory control, and the effects of pharmacological interventions.

In comparison, there are fewer studies on HIE, CP, and Ep, reflecting unique clinical and methodological challenges for these populations, such as participant recruitment, adherence to experimental protocols, and the need for specialized clinical environments. HIE studies frequently require constant monitoring in neonatal intensive care units, while CP studies are often complicated by motor impairment issues that complicate basic task-based paradigms. Of the 72 included studies, 2 examined multiple disorders and were classified according to their primary diagnostic focus.

#### 3.2.2. Temporal and Geographic Distribution

**Figure 2:**
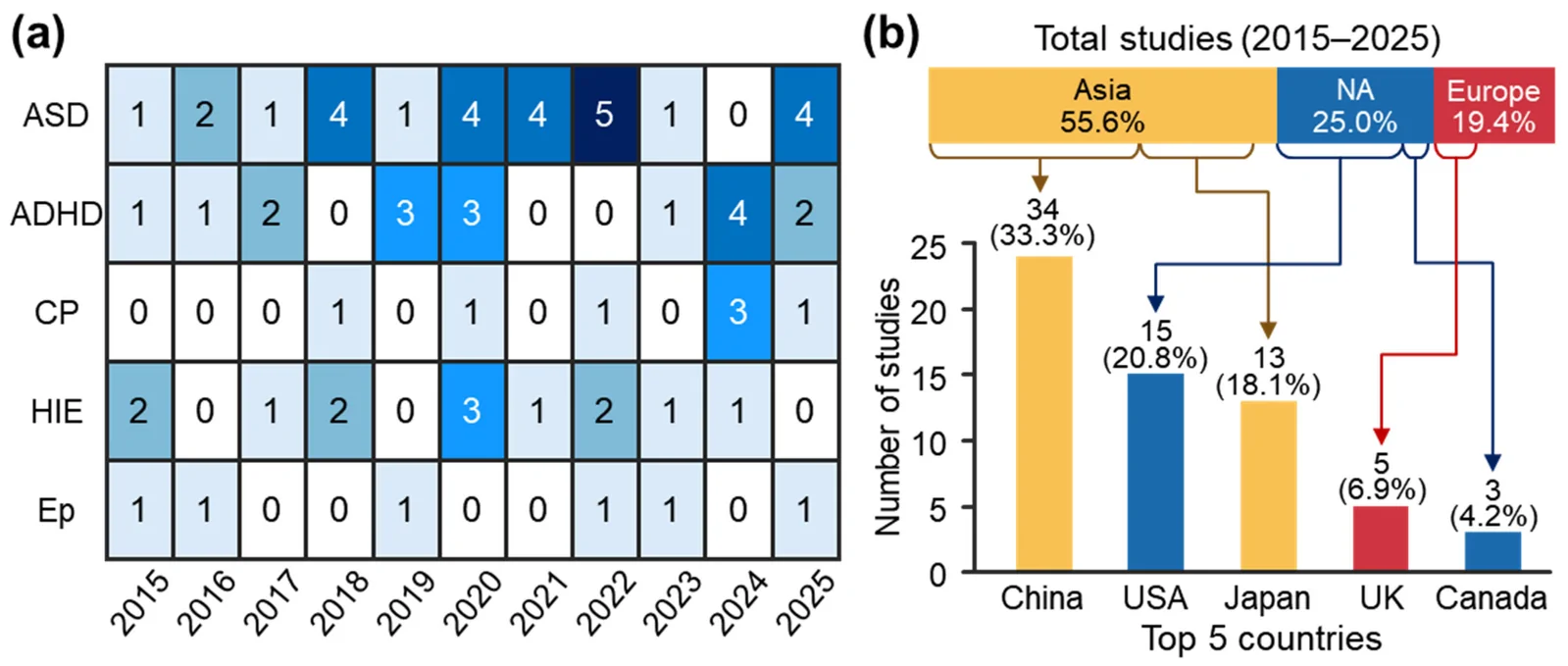
Global trends in fNIRS studies over the past decade (2015–2025). (**a**) Heatmap representing the annual distribution of fNIRS studies across five major pediatric neurodevelopmental disorders. (**b**) Geographic distribution of studies, indicating a predominant contribution from Asia, followed by North America and Europe. fNIRs, functional near-infrared spectroscopy; ASD, autism spectrum disorder; ADHD, attention-deficit/hyperactivity disorder; CP, cerebral palsy; HIE, hypoxic–ischemic encephalopathy; Ep, epilepsy; NA, North America; USA, United States of America; UK, United Kingdom.

The published studies spanned from 2015 to 2025, with a significant increase in publication frequency starting around 2018. The studies mostly contained ADHD and ASD, which showed continued growth across the decade. ADHD-focused studies gradually increased, with spikes around 2019–2020 and 2024, which corresponded with wider use of task-based and resting-state paradigms. Similarly, ASD studies expanded after 2018, showcasing a higher emphasis on communicative, social, and executive systems. In contrast, studies that included CP and HIE were limited and inconsistent, primarily reflecting motor function- and injury-focused investigations and the difficulties of neonatal and pediatric imaging. Ep studies were comparatively limited and heterogeneous, often serving as a pathophysiological comparator rather than a primary neurodevelopmental condition (Figure 2a). This uneven distribution of research studies supports the need for a system-level transdiagnostic synthesis to enable meaningful comparisons despite disparities in research density.

Geographically, the studies were compiled from 15 countries across Asia, Europe, and North America. The Asia-Pacific region contributed the largest proportion, led by China (*n* = 24; 33.3%) and Japan (*n* = 13; 18.1%), which together accounted for over half of all included publications (51.4%). North American contributions were primarily from the United States (*n* = 15; 20.8%) and Canada (*n* = 3; 4.2%). European studies were distributed across several countries, including the United Kingdom (*n* = 5; 6.9%), Germany (*n* = 2; 2.8%), and single contributions from France, Ireland, Italy, the Netherlands, Portugal, Spain, and Turkey (each *n* = 1; 1.4%). Additional Asian studies came from Singapore (*n* = 2; 2.8%) and South Korea (*n* = 1; 1.4%). This geographical pattern conveys both the technological development and clinical research infrastructure in East Asia, especially in China and Japan, where fNIRS technology is widely developed and utilized in pediatric neuroimaging. The high output from Asian institutions directly aligns with large-scale population health initiatives and research funding priorities that are deeply focused on neurodevelopmental disorders (Figure 2b).

#### 3.2.3. Study Design and Sample Characteristics

Most studies administered case–control designs, comparing clinical groups with typically developing or healthy control groups. These designs enabled direct comparison of hemodynamic responses across multiple cognitive and motor tasks. Cohort designs were used in 13 studies (18.1%), particularly in the HIE literature, where longitudinal monitoring is common due to the nature of neonatal intensive care. Randomized controlled and clinical trials were restricted to the ADHD literature (*n* = 5; 6.9%), indicating a narrowed focus on pharmacological treatment effects, especially methylphenidate’s impact on prefrontal activation during attention and executive function tasks. Remaining studies used cross-sectional observational designs. Sample sizes varied widely across studies, ranging from 14 to 207 participants (median = 45).

**Figure 3:**
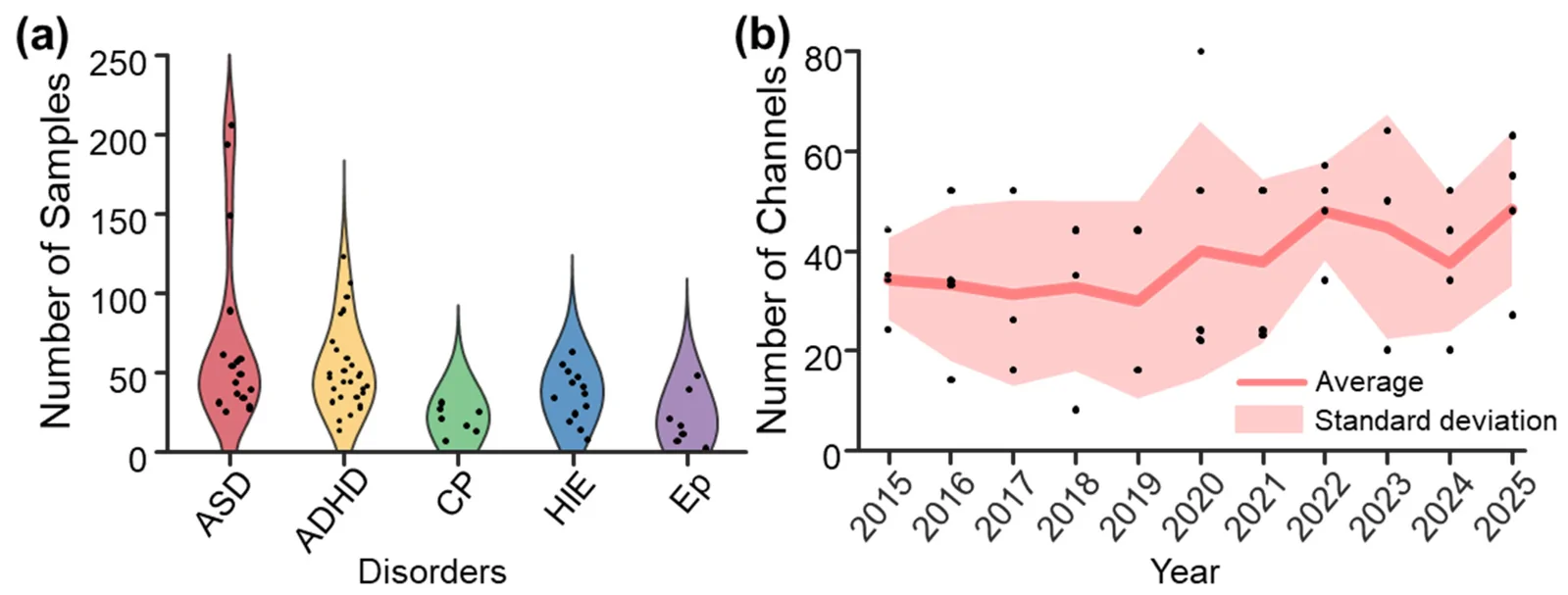
Research trends in fNIRS studies across neurodevelopmental disorders. (**a**) Violin plots showing the distribution of sample sizes across conditions, with individual studies overlaid. The width showcases the density and variability of sample sizes within each group. (**b**) Study-level scatter plot of fNIRS channel counts by year with an overlaid trend line (average) and shaded variability band (standard deviation). The plot signifies temporal variation and channel counts spread across the studies. fNIRS, functional near-infrared spectroscopy. ASD, autism spectrum disorder; ADHD, attention-deficit/hyperactivity disorder; CP, cerebral palsy; HIE, hypoxic–ischemic encephalopathy; Ep, epilepsy.

Studies that researched ADHD and ASD mostly contained larger and more variable sample sizes, with median values of 60 and 47, respectively (Figure 3a). However, CP, HIE, and Ep typically involved smaller cohorts, with median values of 26, 35, and 23, respectively. This pattern likely reflects differences in disorder prevalence, clinical feasibility, and recruitment constraints across conditions. Due to ADHD and ASD being more common disorders with specific diagnostic criteria and bigger patient pools, these studies contributed to larger study samples. Conversely, HIE and CP created unique challenges due to their lower frequency, the severity of clinical presentations requiring intensive medical care, and practical difficulties with neonates, infants, or children who have extensive motor impairments.

Participant age ranges varied considerably across disorders, consistent with differences in typical onset, diagnosis, and clinical course. ADHD studies encompassed both pediatric (4–18 years) and adult (up to 57 years) samples, reflecting its lifespan presentation and interest in developmental persistence. ASD research ranged from early childhood to adulthood (2–45 years), with most studies focusing on school-age children, when social and communication difficulties are more evident in structured environments. CP investigations were limited to pediatric populations (3–18 years), aligning with key periods of motor development and intervention. HIE studies concentrated on neonates and infants (0–24 months), consistent with their perinatal etiology and the importance of early identification. Ep samples included children and adults (5–50 years), reflecting the variable age of onset and chronic seizure disorders.

#### 3.2.4. fNIRS Instrumentation and Configuration

A wide range of fNIRS systems with differing channel densities (2–80 channels) were utilized in studies (Figure 3b). Channel counts varied over time, with an evident spike in 2020 (80 channels), which corresponds to a high-density whole-head system reported in an individual study. Hitachi ETG series devices (ETG-100, ETG-4000, ETG-4100, ETG-7100) were the most used tools, reported in approximately 40% of studies. Wavelength pairs were usually 695/830 nm or 760/850 nm, while HIE studies mainly used clinical cerebral oximetry devices (e.g., INVOS systems) with lower channel counts (2–20 channels), suitable for continuous bedside monitoring.

#### 3.2.5. Task Paradigms and Experimental Approaches

**Figure 4:**
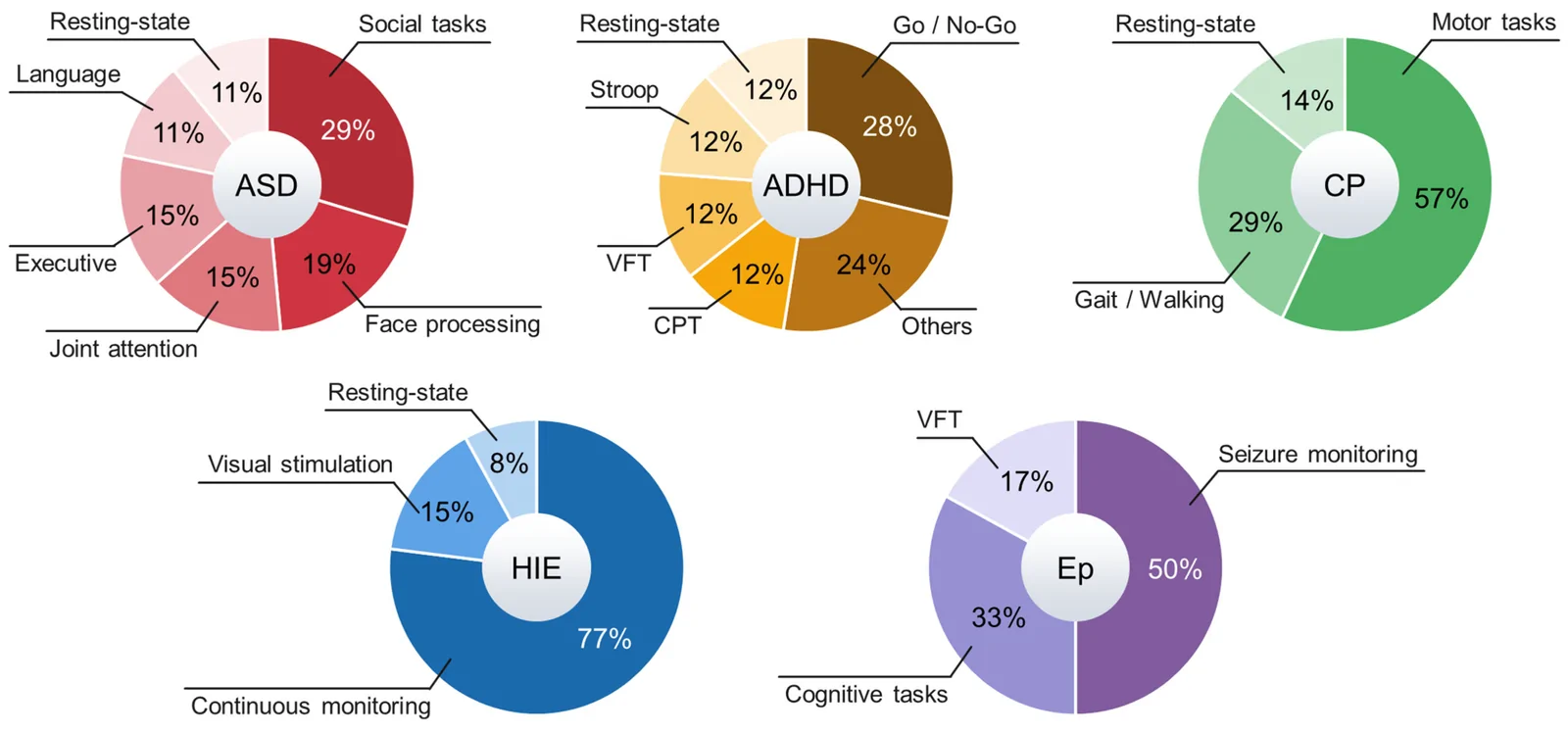
Task paradigms distribution across neurodevelopmental disorders. Pie charts illustrate the proportion of experimental paradigms used across the conditions. ASD, autism spectrum disorder; ADHD, attention-deficit/hyperactivity disorder; CP, cerebral palsy; HIE, hypoxic–ischemic encephalopathy; Ep, epilepsy; CPT, continuous performance task; VFT, verbal fluency tasks.

The task paradigms used in studies showed considerable heterogeneity, reflecting variations in the affected cognitive and behavioral domains in each disorder (Figure 4). ADHD studies used various cognitive tasks, with go/no-go paradigms (29%), stroop tasks (12%), verbal fluency tasks (12%), and continuous performance tasks (12%) being most common, as well as targeting continuous attention and response inhibition. Furthermore, ASD research exhibited the greatest diversity in task selection, with social cognition tasks (30%), face processing paradigms (19%), joint attention tasks (15%), and executive function assessments (15%), thereby emphasizing the core social-pragmatic difficulties characteristic of the disorder. Language tasks (11%) and resting-state recordings (11%) were also frequently used in ASD research.

In comparison, HIE and CP studies showed more focused task sets, showing their main motor and developmental concerns. CP research primarily used motor tasks (57%) and gait/walking assessments (29%), directly addressing core motor impairments in this condition. Meanwhile, HIE research relied extensively on continuous monitoring approaches (77%) for brain injury assessment and prognostication in the neonatal period, supplemented by visual stimulation patterns (15%) and resting-state recordings (8%). Ep studies concentrated on seizure detection (50%), cognitive task performance (33%), and verbal fluency tasks (17%), targeting both ictal activity and interictal cognitive functioning (Figure 4).

Overall, these findings emphasize the significant heterogeneity in fNIRS methodology across neurodevelopmental conditions. Study designs, sample characteristics, and task paradigms were closely tailored to disorder-specific clinical presentations, developmental stages, and research inquiries. Although this specialization yields unique clinical questions, it poses challenges for cross-disorder comparisons and synthesis, highlighting the importance of considering contextual ideals when interpreting broader fNIRS references in neurodevelopmental literature.

### 3.3. Cross-Condition Cortical Region Involvement

To enable meaningful comparisons across disorders, cortical regions reported in the selected studies were systematically organized according to standard anatomical nomenclature. This section highlights patterns of convergence and overlap among the five neurodevelopmental conditions.

#### 3.3.1. Universal and Broad Convergence in Prefrontal Systems

Across the five investigated conditions, the prefrontal cortex emerged as the sole cortical region investigated in all disorder categories, representing a unique anatomical convergence point within the fNIRS literature. This consistent focus underscores the prefrontal cortex’s central role in executive function, attention regulation, behavioral control, and cognitive processing, which are domains commonly disrupted across ASD, ADHD, CP, HIE, and Ep. Within this region, subareas assessed included the dorsolateral prefrontal cortex (ASD, ADHD), the medial prefrontal cortex (ASD, ADHD), the ventrolateral prefrontal cortex (ADHD), and the general prefrontal cortex across all disorders.

Beyond the prefrontal cortex, three regions exhibited broad convergence, being examined in four of five conditions (4/5; 80%): the parietal cortex (ASD, ADHD, CP, HIE), precentral gyrus/motor cortex (ASD, ADHD, CP, Ep), and temporal cortex (ASD, ADHD, HIE, Ep). This distribution reflects shared research priorities, including attention networks (parietal), motor systems (precentral), and language or social-cognitive networks (temporal). Notably, each region displayed condition-specific exclusions: parietal cortex was not targeted in Ep studies, which emphasized seizure-related networks; motor cortex was excluded in HIE research, which focused on global injury assessment; and temporal cortex was not assessed in CP, consistent with the disorder’s predominant motor phenotype. Importantly, the concept was to report the frequency rather than effect size or statistical significance, highlighting research focus rather than direct comparisons of neural dysfunction (Table S1).

#### 3.3.2. Moderate and Limited Convergence Patterns

Moderate convergence (3/5; 60%) was observed for the inferior frontal gyrus (ASD, ADHD, Ep). The involvement of the inferior frontal gyrus finding likely reflects shared interests in language processing, response inhibition, and cognitive control, whereas premotor cortex coverage underscores motor planning and coordination deficits in motor-relevant disorders, although Ep studies did not focus on this region. Limited convergence (2/5 conditions; 40%) was investigated for motor cortex (CP, HIE) and several other regions including specialized prefrontal subdivisions (dorsolateral, medial, frontopolar, middle frontal gyrus in ASD and ADHD), temporal subregions (middle and superior temporal gyrus in ASD and Ep), occipital cortex (ASD and HIE), sensory regions (postcentral gyrus in ASD and Ep; sensorimotor cortex in CP and Ep), and parietal association areas (supramarginal gyrus in ASD and ADHD). These patterns highlight pairwise alignment across functional domains: ASD-ADHD convergence in executive function, ASD-Ep convergence in language networks, and CP-Ep convergence in motor-related areas indicate overlapping interest in movement disorders. Eight regions were uniquely reported in a single disorder (1/5; 20%): angular gyrus, superior frontal gyrus, and ventrolateral prefrontal cortex (ADHD); inferior parietal lobule, superior temporal sulcus, temporoparietal junction, and visual association cortex (ASD); and frontoparietal cortex (HIE). Six of these eight unique regions were within ASD research, reflecting its emphasis on social perception networks (temporoparietal junction, superior temporal sulcus), visual processing (visual association cortex), and posterior association cortices (inferior parietal lobule). HIE’s unique frontoparietal focus aligns with the distributed network assessment of global injury.

#### 3.3.3. Implications for Transdiagnostic and Condition-Specific Research

These patterns support both transdiagnostic and disorder-specific perspectives. The selected fNIRS studies consistently emphasized frontal regions, suggesting that executive and regulatory networks can be a common target of fNIRS. This convergence aligns with evidence that prefrontal networks underlie cognitive control processes affected across disorders, from attention deficits in ADHD to social-cognitive impairments in ASD, motor planning difficulties in CP, and executive dysfunction associated with Ep. Divergence beyond the prefrontal cortex, particularly the disorder-specific targeting of posterior association cortices in ASD and selective inclusion of temporal, parietal, and motor regions, reflects appropriate alignment with disorder phenotypes.

The motor cortex was examined in four conditions and was absent only in HIE, illustrating shared interest in motor systems but nuanced targeting, as HIE research prioritizes global injury assessment over focal motor evaluation. The temporal cortex was assessed in four conditions, absent in CP, while parietal cortex coverage across the same conditions excluded Ep, highlighting convergence within functional domains (language/social cognition vs. attention/sensorimotor integration). This convergence occurs within functional domains rather than universally: language and social cognition disorders (ASD, ADHD, HIE, Ep) share temporal emphasis, whereas attention and sensorimotor integration disorders (ASD, ADHD, CP, HIE) share parietal emphasis. Methodologically, the universal focus on the prefrontal cortex suggests that this region is particularly well-suited as a target for future transdiagnostic fNIRS studies aimed at identifying shared neural mechanisms. Across the corpus of studies, 24 distinct regions were examined, of which 33% were investigated in a limited or disorder-specific manner. This demonstrates that fNIRS research can capture both disorder-specific neural signatures and sufficient cross-condition overlaps to support comparative and dimensional analyses. This balance highlights that shared circuits (prefrontal executive systems) and disorder-specific networks (such as ASD posterior social perception systems) can be examined simultaneously within a unified neurobiological framework.

#### 3.3.4. Anatomical Context and Spatial Interpretation

**Figure 5:**
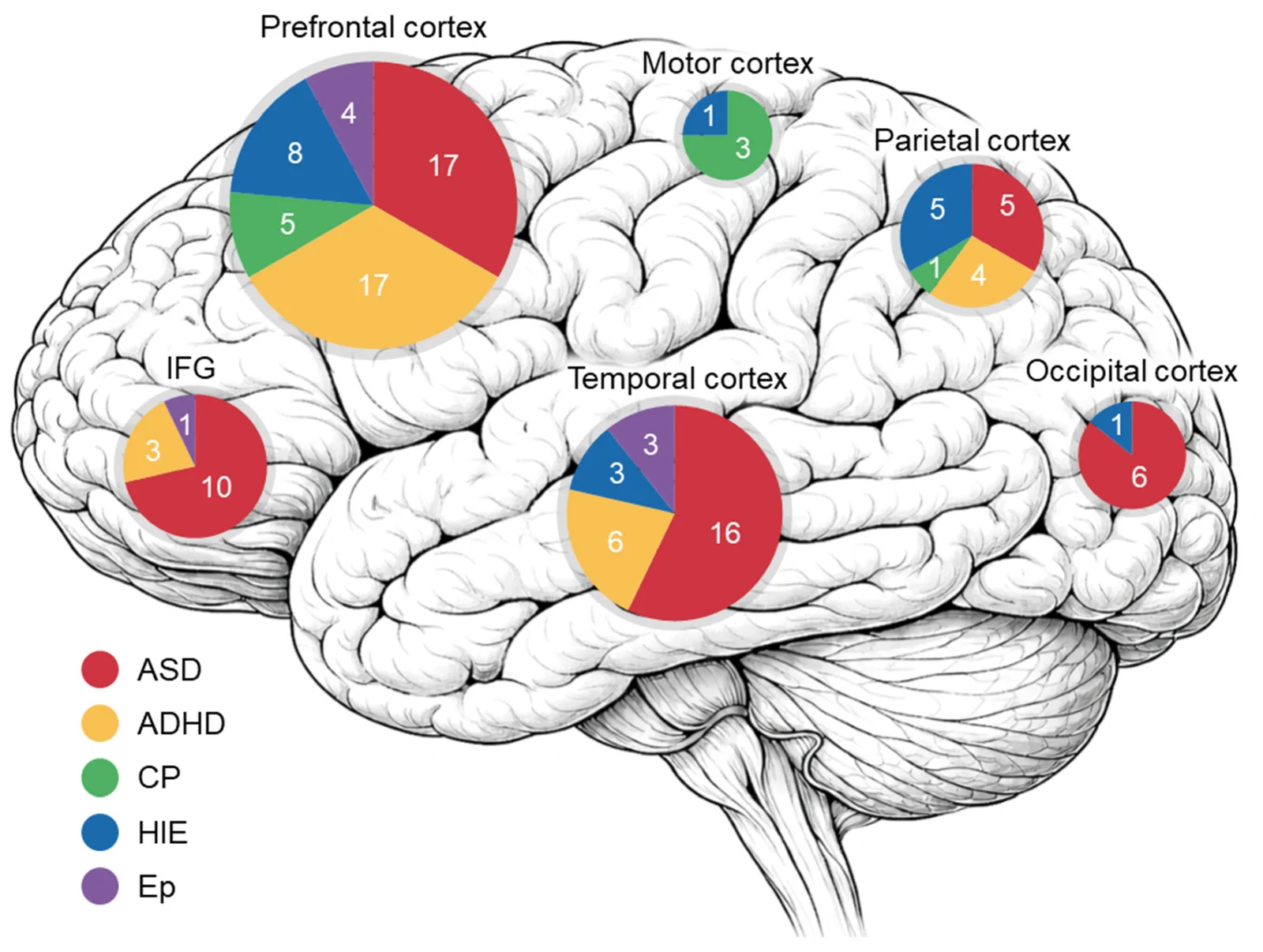
Cortical regions reporting patterns across neurodevelopmental disorders in fNIRS studies. Lateral hemisphere view with pie charts representing the number of studies per region within each condition, with the prefrontal cortex most frequently explored. Disorder-specific foci are presented for IFG (ASD) and motor cortex (CP). fNIRS, functional near-infrared spectroscopy; ASD, autism spectrum disorder; ADHD, attention-deficit/hyperactivity disorder; CP, cerebral palsy; HIE, hypoxic–ischemic encephalopathy; Ep, epilepsy; IFG, inferior frontal gyrus.

The system-level patterns are anatomically contextualized in Figure 5. Regions exhibiting the highest degree of cross-condition overlap are primarily concentrated in frontal and frontoparietal areas, notably within the prefrontal cortex and neighboring motor regions. Temporal regions show moderate cross-disorder convergence, whereas posterior cortical areas are less consistently represented. This brain schematic indicates that convergence across disorders is most pronounced within frontal control networks. In contrast, divergence is more apparent in posterior, sensorimotor, and association cortices. This spatial pattern aligns with a transdiagnostic perspective in which frontal systems represent a shared research focus across neurodevelopmental disorders, while condition-specific investigations of other cortical regions reflect differences in developmental constraints, injury profiles, or disorder-specific functional phenotypes.

#### 3.3.5. Role of Epilepsy as a Comparative Pathophysiological Framework

Ep was included in this cross-condition analysis to provide a pathophysiological context for cortical network involvement. Unlike the four neurodevelopmental disorders (ASD, ADHD, CP, HIE), Ep is defined by dynamic, state-dependent reorganization of neural networks linked to seizure activity, rather than by persistent developmental changes [82]. Within this body of work, Ep studies were predominantly focused on the prefrontal cortex, examining executive control during interictal periods, the temporal cortex, for language mapping and localization of seizure foci, and the motor cortex, associated with seizure propagation pathways. In contrast, parietal cortex and posterior association regions, which are frequently investigated in ASD and ADHD research, were not consistently assessed in Ep studies.

Although prefrontal and temporal regions overlap anatomically between Ep and the neurodevelopmental disorders, the underlying mechanisms differ substantially [88]. Executive dysfunction observed in ASD and ADHD results from developmental alterations in neural circuits, whereas in Ep, it may emerge from seizure-related disruption or compensatory network reorganization. Temporal cortex involvement also reflects distinct processes across disorders: in Ep, it primarily relates to seizure propagation [89] and surgical planning, whereas in ASD and HIE, it reflects social communication impairments and diffuse cortical injury [90]. Consequently, while there is spatial overlap at the regional level, functional equivalence should not be concluded. For this reason, Ep findings were excluded from analyses of developmental trajectories in subsequent sections and were instead used as an anatomical and functional comparator to clarify how similar cortical regions can participate in qualitatively different neural processes across stable developmental conditions versus dynamic seizure-related pathophysiology.

### 3.4. Disorder-Specific Cortical Profiles

#### 3.4.1. Autism Spectrum Disorder

Studies investigating ASD have revealed a broad cortical profile, with pronounced involvement of temporal, temporoparietal, and inferior frontal regions, as well as frontal regions [18,21,24,26,30,31,32,33,34,35,40,42,44,45]. Altered engagement of social brain networks, including the superior temporal sulcus, temporoparietal junction, and inferior frontal gyrus, was frequently reported during social cognition, language, and interaction-based paradigms. Resting state and hyperscanning studies further identified disrupted interhemispheric connectivity and reduced interpersonal neural synchrony, emphasizing altered network coordination rather than focal dysfunction [27,29,36,39,40]. Collectively, ASD findings reflect pervasive alterations in social–communicative and integrative cortical systems. Study details are provided in Table 1.

#### 3.4.2. Attention-Deficit/Hyperactivity Disorder

Across studies of ADHD, the most prominent cortical feature was altered engagement of prefrontal executive systems [46,49,51,52,53,55,56,57]. Task-based research consistently highlighted involvement of the dorsolateral and inferior frontal regions during paradigms that demand inhibitory control and sustained attention, with several studies noting atypical hemispheric patterns, often characterized by changes in right prefrontal activation. Analyses focusing on connectivity revealed disrupted organization within frontoparietal and default mode networks, indicating inefficient integration across large-scale neural systems [60,61]. Additionally, pharmacological investigations [46,47] demonstrated that prefrontal activation can be modulated by treatment, underscoring fNIRS’s sensitivity to neural changes resulting from intervention. Comprehensive study characteristics are provided in Table 2.

#### 3.4.3. Cerebral Palsy

In contrast to the other disorders, studies of CP were predominantly characterized by involvement of sensorimotor cortical regions [63,64,65,66]. fNIRS investigations commonly reported increased or bilateral activation in the motor and somatosensory cortices during tasks involving movement and gait, which is frequently interpreted as compensatory recruitment following early brain injury. Additional findings included reduced interhemispheric connectivity within motor networks and elevated recruitment of frontal regions during motor tasks, suggesting that movement planning in CP imposes greater cognitive demands. Collectively, these results emphasize that CP is defined primarily by injury-driven reorganization of motor networks, rather than by disruptions in executive or social brain systems [67,68,69]. Detailed study characteristics are provided in Table 3.

#### 3.4.4. Hypoxic–Ischemic Encephalopathy

HIE studies were distinct from the other conditions in both their methodological approaches and observed cortical profiles, focusing primarily on neonatal cerebral oxygenation, autoregulatory processes, and neurovascular coupling rather than on task-evoked cortical activation [71,72,74,75,79]. Frequently reported findings included altered cerebrovascular regulation, asymmetry in interhemispheric oxygenation, and disruptions in long-range connectivity, reflecting widespread cortical vulnerability associated with global brain injury [78,80]. In older infants and survivors, network analyses demonstrated extensive hypoconnectivity, with relative preservation of short-range connections, consistent with adaptive cortical reorganization following hypoxic–ischemic insult [70,73,78,82]. Overall, these results underscore a cortical profile in HIE that is primarily physiological rather than task-specific. Detailed study characteristics are summarized in Table 4.

#### 3.4.5. Epilepsy

Studies of Ep revealed heterogeneous, state-dependent patterns of cortical involvement that varied according to seizure type and focus [83,86]. fNIRS investigations frequently reported focal hemodynamic changes in frontal and temporal regions during seizure events, accompanied by alterations in hemispheric organization within language and cognitive networks in pediatric populations [83,84,85]. In contrast to neurodevelopmental disorders, cortical changes associated with Ep were dynamic and context-specific, reflecting transient network reorganization rather than enduring developmental disruption [86]. For this reason, Ep findings were incorporated as a pathophysiological comparator, providing additional context to interpret cortical lateralization and dynamics across the set of selected disorders. Detailed study characteristics are provided in Table 5.

### 3.5. Comparative Synthesis of Cortical Region Reporting Patterns

Across the conditions studied, four cortical features consistently emerged that extend beyond individual diagnoses: (1) altered engagement of prefrontal executive systems, particularly within the dorsolateral and inferior frontal cortices during tasks with high cognitive demands (ASD, ADHD, Ep); (2) disruptions in large-scale network organization, evidenced by reduced interhemispheric coupling and diminished long-range connectivity across all disorders, while local connectivity was often maintained or even enhanced in injury-related conditions (CP, HIE); (3) atypical developmental trajectories that deviate from expected age-related maturation patterns; and (4) compensatory recruitment of neural resources in contralateral or adjacent regions, most prominently observed in CP, HIE, and Ep. These transdiagnostic features were apparent despite substantial variability in participant ages, experimental paradigms, and fNIRS instrumentation.

## 4. Discussion

### Overall Interpretation

A systematic review of 72 fNIRS studies across five pediatric conditions (ASD, ADHD, CP, HIE, Ep) revealed consistent reporting of prefrontal cortex patterns, aligning with the Research Domain Criteria (RDoC) framework [5]. This review examined patterns in cortical regions rather than solely activation findings. The emphasis on prefrontal regions is consistent with individual studies that report alterations in prefrontal activation, disrupted connectivity, and compensatory recruitment in various pediatric conditions. This pattern has several clinical implications. First, the prefrontal cortex emerged as the sole region examined in all 5 conditions (5/5; 100%), whereas the parietal and temporal cortices were represented in 4 of the 5 conditions (4/5; 80%). From a lobar analysis, the frontal cortex was studied most extensively, with 29 region reports, while the temporal cortex showed the greatest consistency across conditions (mean 2.25 conditions per region). Second, condition-specific cortical patterns closely corresponded to disorder phenotypes: ASD studies focused on social brain networks, ADHD on prefrontal executive systems, CP on sensorimotor reorganization, HIE on cerebrovascular monitoring, and Ep on state-dependent cortical dynamics. Third, transdiagnostic characteristics were identified, including altered prefrontal engagement, disrupted large-scale connectivity, and compensatory recruitment of neural resources.

The consistent examination of the prefrontal cortex across all conditions underscores its role as a late-maturing, integrative neural hub that is particularly vulnerable to disruption in diverse developmental contexts. Serving as a central node within multiple large-scale networks, including executive control, default mode, and salience networks, dysfunction in the prefrontal cortex can propagate effects across distributed neural systems [88]. This pattern of convergence suggests that prefrontal regions may provide an ideal target for transdiagnostic fNIRS investigations aimed at uncovering shared neural mechanisms and dimensional biomarkers across pediatric neurodevelopmental disorders.

The distributed ASD cortical profile emphasizing temporal, temporoparietal, and inferior frontal regions supports “social brain hypothesis” models [18,20,21,22,24,25,26,27,28,30,31,32,33,34,35,39,41,43,45], with hyperscanning studies revealing reduced interpersonal neural synchrony providing network-level evidence [27,29,36,37,40]. ADHD’s prefrontal concentration during attention tasks supports executive dysfunction models [46,49,51,52,53,56,57,60], while pharmacological studies demonstrating modifiable activation with methylphenidate suggest state-dependent alterations [46,47,53]. CP’s sensorimotor focus with bilateral compensatory activation illustrates injury-driven reorganization [63,64,65,66]. HIE’s cerebrovascular monitoring revealed altered neurovascular coupling and interhemispheric asymmetry [71,72,74,75,79], suggesting effects on fundamental brain physiological processes. Ep’s state-dependent dynamics distinguished it from neurodevelopmental conditions, underscoring the importance of temporal dynamics and network states for understanding pathophysiology [83,86].

Although several cortical regions were reported across multiple disorders, their neurobiological significance differed substantially according to the underlying disease mechanisms. Frontal cortical involvement in ASD and ADHD reflects alterations in social cognition and executive control, respectively, whereas sensorimotor regions are more closely associated with motor network reorganization in CP. Similarly, cortical measurements in HIE primarily represent neurovascular regulation, while temporal and frontal involvement in Ep reflects cortical network reorganization and seizure-related functional adaptation. These observations indicate that similar cortical targets may support distinct neural processes depending on the clinical context.

However, a few gaps limit the immediate clinical translation, as the predominant focus on prefrontal regions may indicate methodological convenience instead of detailed assessments of neural mechanisms. The underexploration of deeper or posterior regions (e.g., occipital cortex: 7 studies, 9.7%) highlights technical limitations in these regions. Additionally, pharmacological studies in ADHD illustrate the potential of fNIRS for monitoring treatment, as its portability allows repeated-measures tracking of neural changes. Still, systematic investigations are needed to establish the relationship between fNIRS-detected activity and functional outcomes before clinical application.

Importantly, the methodological suitability of fNIRS differs across disorders according to the cortical systems and experimental paradigms investigated. In ASD and ADHD, fNIRS is particularly advantageous for monitoring prefrontal activity during cognitive and social tasks because of its tolerance for naturalistic behavioral paradigms. However, due to the limited penetration depth and lower spatial resolution of fNIRS [91], this may restrict deep neural network assessments. In contrast, CP studies benefit from repeated measurements of sensorimotor cortical function during rehabilitation, although movement artifacts remain a significant challenge. HIE primarily exploits the portability of fNIRS for bedside monitoring of neonatal cerebral hemodynamics, whereas Ep studies frequently combine fNIRS with EEG to characterize cortical hemodynamics despite limited sensitivity to deep epileptogenic networks. In addition, differences in instrumentation, probe geometry, optode configuration, wavelength selection, and preprocessing pipelines should be considered when interpreting cortical reporting patterns across disorders, as these methodological variations can substantially influence cortical sensitivity, spatial localization, and reproducibility of reported activation patterns (Note S3) [92,93,94,95,96,97].

fNIRS made it possible to investigate populations and paradigms that are challenging for traditional neuroimaging, including neonates in intensive care (HIE), children with severe motor impairments (CP), and naturalistic social interactions in ASD hyperscanning. Its portability enables bedside monitoring for prognostic assessment and ecological task designs that capture real-world brain function. However, fNIRS is limited to cortical surface measurements, offers lower spatial resolution than fMRI, and faces notable standardization issues, including heterogeneous analysis pipelines, inconsistent channel placement, incomplete methodological reporting, and limited accounting for age-related scalp-to-cortex distances. Sample sizes varied considerably (14–207; median: 45), with smaller cohorts in clinically complex populations raising concerns about statistical power. Due to substantial heterogeneity, this scoping review emphasized descriptive system-level patterns rather than quantitative meta-analysis. The literature was unevenly distributed, with more studies on ADHD and ASD compared to CP, HIE, and Ep. Categorizing data into broad anatomical regions also reduces spatial specificity. Nevertheless, the observation of consistent system-level patterns across diverse methodologies supports the validity of a transdiagnostic framework.

Future studies should prioritize standardized task paradigms and longitudinal designs to better delineate developmental trajectories of cortical function across pediatric disorders. Integrating fNIRS with other modalities, such as EEG or MRI, could improve mechanistic understanding, particularly in conditions involving neurovascular or metabolic disturbances. While fNIRS represents a promising, child-friendly method for assessing brain function, additional validation is required before system-level metrics can be reliably incorporated into clinical decision-making.

## 5. Conclusions

This systematic review underscores both convergent and disorder-specific cortical patterns across pediatric neurodevelopmental and neurological conditions. The prefrontal cortex was the only region assessed across all conditions, highlighting its potential as a transdiagnostic target. Across the 72 included studies, cortical reporting patterns demonstrated varying degrees of cross-condition convergence, while disorder-specific cortical profiles reflected characteristic phenotypes and research priorities. Shared system-level features support dimensional approaches to understanding neurodevelopmental pathophysiology. Our findings further indicate that methodological heterogeneity, inconsistent cortical region reporting, and the uneven distribution of evidence across disorders remain major challenges for cross-study comparison and interpretation. Addressing these limitations through standardized acquisition protocols, reporting practices, and broader cortical coverage will improve the reproducibility and clinical utility of future fNIRS research. Despite these limitations, fNIRS holds substantial promise for advancing both fundamental neurodevelopmental research and clinical applications, providing distinctive insights into cortical function throughout childhood via its accessibility, ecological validity, and ability to capture naturalistic paradigms that are difficult to study with conventional neuroimaging techniques.

## Figures and Tables

**Table 1:** Summary of ASD studies. ASD, autism spectrum disorder; ABB, Adjacent B-B Repetition Sequence; ABC, All-By-Change Syllable Sequence; ADOS, Autism Diagnostic Observation Schedule; ASC, autism spectrum condition; AVM, animated video modeling; DFA, detrended fluctuation analysis; DLPFC, dorsolateral prefrontal cortex; EL, elevated likelihood; F, frontal; FC, functional connectivity; FPC, Fronto parietal cortex; FPA, frontopolar area; HbO, oxygenated hemoglobin; HbR, deoxygenated hemoglobin; HbT, total hemoglobin; HR, high-risk; HRA, high-risk autism; HRI, high-risk infants; IFG, inferior frontal gyrus; IPL, inferior parietal lobule; IPS, interpersonal synchrony; L, left; L-DLPFC, left dorsolateral prefrontal cortex; L-IFG, left inferior frontal gyrus; L-MFG, left middle frontal gyrus; L-PFC, left prefrontal cortex; L-STG, left superior temporal gyrus; LOL, left occipital lobe; LR, low-risk; LRA, low-risk autism; LRI, low-risk infants; LRTCs, long-range temporal correlations; m, months; MFG, middle frontal gyrus; mPFC, medial prefrontal cortex; MTG, middle temporal gyrus; $N_{ch}$, number of channels; $N_{s}$, number of samples; NT, neurotypical; O, occipital; OEMS, observation–execution matching system; P, parietal; PCG, pre/post-central gyrus; PFC, prefrontal cortex; PMC, premotor cortex; R, right; R-DLPFC, right dorsolateral prefrontal cortex; R-IFG, right inferior frontal gyrus; R-MFG, right middle frontal gyrus; R-PFC, right prefrontal cortex; R-TPJ, right temporo-parietal junction; ROL, right occipital lobe; RSFC, resting-state functional connectivity; SampEn, sample entropy; SCFC, spatial complexity of functional connectivity; SCZ, schizophrenia; SD, sleep disorder; SMG, supramarginal gyrus; STG, superior temporal gyrus; STS, superior temporal sulcus; T, temporal; TC, temporal cortex; TD, typically developing; TL, typical likelihood; TPJ, temporo-parietal junction; VAC, visual association cortex; VFT, verbal fluency task; WM, working memory; y, years; >, indicates greater magnitude than; ↑, increase; ↓, decrease; ↓↓, strong decrease/clear reduction.

Ref.	Type	fNIRS		Study Design					Result	
		$N_{ch}$	λ [nm]	$N_{S}$	Age	Task	Region	Measurements	Physiological Alteration	Main Findings
[18]	Case control	34	-	77 ASD, 31 TD	3–6 y	Emotional facial imitation	IFG	HbO	↓ HbO during imitation; ↑ HbO after imitation training	Mirror neuron dysfunction; IFG plasticity
[19]	Case control	14	690, 830	8 ASD, 12 NT	7–36 y	1-back face recognition	Bilateral T, O regions	HbO	↓ R-hemisphere asymmetry	Atypical face-processing asymmetry
[20]	Case control	52	805, 830	25 ASD, 22 TD	7–11 y	Resting state	Bilateral TC	FC, HbO, HbR	↓ RSFC ↑ HbO/HbR fluctuations	Reduced temporal RSFC
[21]	Clinical	26	780, 850	16 HR, 13 LR	5 m	Social/non-social visual stimuli	Bilateral TC, posterior STS-TPJ	HbO, HbR	↓ HbO activation (social stimuli)	Early social-processing atypicality
[22]	Case control	-	695, 830	24 ASD, 24 TD	6–15 y	Go/No-Go	Bilateral IFG, MFG	HbO, HbR	↓↓ Activation in R-IFG/MFG	Right frontal hypoactivation
[23]	Clinical	44	780, 805, 830	46	2–8 y	Watching cartoon	Bilateral PFC, T, O cortex	Efficiency of HbO, HbR, HbT	↓ network efficiency	Reduced network efficiency
[24]	Case control	-	-	13 ASD, 15 SCZ, 18 TD	23–47 y	VFT & face recognition	L-IFG, L-STG, L-MTG	HbO	↓ L-frontotemporal activation (ASD)	Left frontotemporal hypoactivation
[25]	Case control	44	780, 805, 830	29 ASD, 29 TD	4–9 y	Passive cartoon viewing	Bilateral PFC, T, O lobes	HbO, HbR, HbT, SCFC	↑ SCFC in ASD	Aberrant frontal–temporal SCFC
[26]	Case control	44	780, 805, 830	29 ASD, 29 TD	4–9 y	Passive cartoon viewing	Bilateral prefrontal, T, temporo-occipital cortices	LRTCs via DFA on HbO/HbR	↓↓ DFA exponent; ↓↓ HbO (L-T); ↓↓ HbR (Bilateral Temporo-occipital)	Randomized temporal oscillations
[27]	Clinical	24	695, 830	9 HRI, 6 LRI	6–9 m	Live social interaction	Bilateral TC, inferior frontal regions, parietal sites	HbO, FC	↓ HbO: social interaction in HR ↓ FC: U-shaped connectivity	Early social network atypicality
[28]	Case control	52	695, 830	20 ASD, 19 TD	8–12 y	n-back WM	PFC	HbO, FC	↑ PFC activation ↓ R-frontal connectivity	Executive network inefficiency
[29]	Case control	22	690 830	16 ASD	7–10 y	2-person key-press task	PFC	Joint HbO shift	↑ Parent–child neural synchrony ↓ Synchrony (greater ASD severity)	Altered parent–-child synchrony
[30]	Case control	24	695 830	14 ASD, 17 TD	10–12 y	Watch, Do, together	MIFG, MSTG, IPL	HbO, HbR	↓ MIFG/MSTG ↑ IPL	Social-motor integration deficit
[31]	Case control	44	780, 805, 830	25 ASD, 22 TD	8–11 y	Resting state	IFG, TC	HbO	SampEn magnitude: L-IFG > R-IFG > TC	Reduced neural complexity
[32]	Case control	48	780 805 830	70 ASD, 40 TD	6–16 y	Resting state	mPFC, bilateral TPJ, L-MFG	HbO	TPJ U-shaped, MFG decreasing with age, mPFC typical	Abnormal hemispheric lateralization
[33]	Case control	52	695 830	15 ASD, 15 TD	11–13 y	Observe sway, Solo sway, Face-to-face	IFG, STS	HbO, HbR	↓ IFG & ↓ STS activation during IPS	IFG/STS social biomarkers
[34]	Clinical	24	695 830	18 LRA, 14 HRA	6 m	Listening to ABB & ABC	Anterior (F, T) & Posterior (P, O)	HbO	↓ anterior activation (HRA+) ↑ right-posterior activation (HRA−)	Early speech-network atypicality
[35]	Case control	-	780 805 830	25 ASD, 22 TD	8–10 y	Resting state	Bilateral TC	RSFC	↓ Interhemispheric temporal RSFC	Reduced temporal connectivity
[36]	Case control	22	-	41 TD, age-matched: 18 ASD + 18 TD	8–18 y	Cooperative vs. competitive button press	DLPFC, FPC	HbO, HbR	↓ motor synchrony in PFC	Impaired interpersonal synchrony
[37]	Cross sectional	57	760 850	65 EL, 59 TL, 59 EL, 38 TL	5 m, 10 m	Resting state	F & T lobes	Local/nodal efficiency,	↑ local/nodal efficiency (5 m) no major difference (10 m)	Pre-symptomatic network atypicality
[38]	Case control	52	695 830	22 ASD, 24 TD	8–12 y	n-back WM	Bilateral/medial PFC	HbO	↓↓ R-hemisphere (higher WM load) L-PFC: no change	Right-PFC working-memory dysfunction
[39]	Case control	-	-	45 ASD, 53 NT	4–6 y	Video: robotic vs. human interaction	L-DLPFC, R-DLPFC	HbO	↓ R-DLPFC (robot vs. human in ASD), ↓ L-DLPFC linked to ADOS severity	Abnormal DLPFC social response
[40]	Case control	52	695 830	15 ASC, 15 TD	6–17 y	Lincoln Log dyadic building game	IFG, MFG, STS, IPL, PCG	HbO, HbR, OEMS activation	↓ STS ↑ IPL activation in ASC	Altered joint-action processing
[41]	Case control	52	-	21 ASD, 23 TD	8–12 y	VFT	PFC	HbO, HbR, FC	↓↓ PFC + FC in ASD	Frontal executive dysfunction
[42]	Case control	-	-	25 ASD, 22 TD	8–11 y	Resting state	Bilateral F, T cortices	HbO, HbR	↓ L-frontal lobe	Disrupted frontal HbO/HbR networks
[43]	Case control	63	-	15 ASD, 15 TD	3–7 y	Rest + AVM	R-DLPFC, L-DLPFC, mPFC, ROL, LOL, IPL, PMC	HbO, FC	↑ R-DLPFC/IPL, mPFC during AVM	Impaired resting connectivity
[44]	Case control	63	-	29 ASD + SD, 29 ASD + SD, 30 TD	4–9 y	Resting state	SMG, IFG, FPA, DLPFC, VAC	RSFC	↓ L-IFG connectivity (social-related) ↑ Sleep disturbance (altered R-IFG FC)	Sleep-related network disruption
[45]	Case control	48	690 830	15 ASD, 16 NT	3–6 y	Live/recorded language	Bilateral IFG, MFG, STG, MTG, TPJ	HbO	↑ R-TPJ activation (NT) No change (ASD)	Impaired social-language processing

**Table 2:** Summary of ADHD studies. ADHD, attention-deficit/hyperactivity disorder; ADHD-C, ADHD combined type; ADHD-I, ADHD inattentive type; ADHD + ASD, ADHD with autism spectrum disorder; ADHD + SLD, ADHD with specific learning disorders; ALFF, amplitude of low-frequency fluctuation; ANG, angular gyrus; ASD, autism spectrum disorder; ATX, atomoxetine; CANTAB, Cambridge Neuropsychological Test Automated Battery; CPT, continuous performance task; DLPFC, dorsolateral prefrontal cortex; F, frontal; FC, functional connectivity; HA, hyperactive; HbO, oxygenated hemoglobin; HbR, deoxygenated hemoglobin; HbT, total hemoglobin; HC, healthy controls; IFG, inferior frontal gyrus; IFC, inferior frontal cortex; L, left; L-DLPFC, left dorsolateral prefrontal cortex; L-PFC, left prefrontal cortex; L-PPC, left posterior parietal cortex; L-VLPFC, left ventrolateral prefrontal cortex; MFG, middle frontal gyrus; mPFC, medial prefrontal cortex; MPH, methylphenidate; $N_{ch}$, number of channels; $N_{s}$, number of samples; O, occipital; P, parietal; PFC, prefrontal cortex; PPC, posterior parietal cortex; PrCG, precentral gyrus; R, right; R-DLPFC, right dorsolateral prefrontal cortex; R-IFG, right inferior frontal gyrus; R-MFG, right middle frontal gyrus; R-PFC, right prefrontal cortex; R-PPC, right posterior parietal cortex; R-SMG, right supramarginal gyrus; R-temporal, right temporal; R-VLPFC, right ventrolateral prefrontal cortex; RCT, randomized controlled trial; RSFC, resting-state functional connectivity; SFC, superior frontal cortex; SFG, superior frontal gyrus; SMG, supramarginal gyrus; SST, stop signal task; T, temporal; TC, temporal cortex; TCO, two-choice oddball task; TD, typically developing; TDC, typically developing controls; VFT, verbal fluency task; VLPFC, ventrolateral prefrontal cortex; WM, working memory; y, years; λ, wavelength; ↑, increase; ↓, decrease.

Ref.	Type	fNIRS		Study Design					Result	
		$N_{ch}$	λ [nm]	$N_{S}$	Age	Task	Region	Measurements	Physiological Alteration	Main Findings
[46]	Case Control	24	780, 830	12 ADHD, 14 TD	6–13 y	CPT	DLPFC, VLPFC	HbO	Pre: ↓ frontal Post-ATX: ↑ R-DLPFC	ATX normalized R-DLPFC during CPT
[47]	RCT	-	-	30 ADHD, 20 HC	6–12 y	SST	F (R-IFC, L-IFC)	HbO	Baseline: ↓ R-IFC Post-MPH: ↑ frontal activation	MPH normalized activation; L-IFC predicted response
[48]	Case control	-	-	30 ADHD, 35 TD	8–11 y	CANTAB spatial WM	F (frontal pole, bilateral lateral PFC)	HbO	↓ Frontal activation trajectory (ADHD)	Abnormal developmental trajectory
[49]	Case control	52	695, 830	14 ADHD, 15 TD	6–9 y	Go/No-Go	L-PFC	HbO	↓ L-frontopolar activation (ADHD)	Impaired frontal executive engagement
[50]	Case-control	16	-	12 ADHD, 14 TD	8–11 y	Flanker task	SFC	HbO	↑ Superior frontal activation (ADHD)	Greater SFC activation during conflict
[51]	Case control	16	730, 850	23 ADHD, 21 controls	7–12 y	Auditory oddball	R-PFC	HbO	↓ Prefrontal activation (ADHD)	Impaired frontal attentional processing
[52]	Case control	44	695, 830	21 ADHD, 21 controls	7–9 y	Go/No-Go	IFG, MFG, SMG, ANG	Task-based FC	↓ Frontal activation (ADHD) ↑ R-temporal (HA)	Reduced frontoparietal network engagement
[53]	RCT	44	695, 830	21 ADHD, 11 ASD	7–9 y	Go/No-Go	MFG, IFG, PrCG, SMG, ANG	HbO, frontoparietal network	↑ frontoparietal (ADHD) ↓ motor activation post-MPH (ADHD + ASD)	Comorbidity-dependent medication effects
[54]	Case control	16	770, 840	67 ADHD, 140 TD	8–11 y	Reverse Stroop	R-PFC, mPFC, L-PFC	HbO	↓ R/M-PFC ↑ L-PFC (compensatory)	L-PFC compensatory role
[55]	Case control	44	695, 830	20 ADHD, 15 HA, 27 TDC	6–13 y	Go/No-go	F in ADHD; R-temporopolar in HA	HbO	↓ ADHD (frontal), HA (R-temporal)	Distinct neural mechanisms
[56]	Case control	80	-	30 ADHD, 30 HC	7–12 y	Resting state	F, T, P, O cortices	RSFC, network efficiency	↓ FC ↓ global efficiency in ADHD	Widespread network hypoconnectivity
[57]	Case control	52	695, 830	34 ADHD-C, 52 ADHD-I, 24 HC	6–13 y	Resting state	mPFC, L-PPC, R-PPC, L-DLPFC, R-DLPFC, L-VLPFC, R-VLPFC, TC	ALFF, RSFC	↑ ALFF ↓ FC in ADHD	ADHD-C stronger abnormalities than ADHD-I
[58]	Case control	44	695, 830	30 ADHD, 30 ADHD + SLD, 30 TD	6–12 y	TCO	Bilateral PFC (R-MFG/IFG border)	HbO	TD > ADHD > ADHD + SLD (progressive ↓)	Graded R-PFC reduction with comorbidity
[59]	Case control	40	730, 808, 850	52 ADHD, 34 HC	4–9 y	GO/No-go	Whole-brain (MFG/SFG, R-SMG, L-PCG)	HbO	↓ ADHD (widespread hypoactivation)	PFC hypoactivation; motor hyperconnectivity
[60]	Case control	-	-	14 ADHD, 14 TD	N/A	CPT	R-DLPFC	HbO	↓ R-DLPFC activation (ADHD) ↑ R-DLPFC (TD)	Weakened frontal–attentional coupling
[61]	Case control	52	695, 830	75 HC, 75 naïve ADHD, 45 medicated ADHD	21–57 y	VFT	F, T regions	HbO	↓ Frontotemporal activation (ADHD)	L-lateralized hypoactivation
[62]	Case control	52	695, 830	75 HC, 75 naïve ADHD	36–37 y	VFT	PFC, TC	Inter-channel FC, HbO, HbR, HbT	↓ Hypoconnectivity (ADHD)	Network disruption correlates with symptoms

**Table 3:** Summary of CP studies. CP, cerebral palsy; FC, functional connectivity; F, frontal; HbO, oxygenated hemoglobin; HCP, hemiplegic cerebral palsy; L, left; L-MC, left motor cortex; L-PFC, left prefrontal cortex; LSUT, lateral step-up test; MC, motor cortex; $N_{ch}$, number of channels; $N_{s}$, number of samples; P, parietal; PFC, prefrontal cortex; R, right; R-MC, right motor cortex; R-PFC, right prefrontal cortex; RAGT, robot-assisted gait training; SMC, sensorimotor cortex; TD, typically developing; y, years; λ, wavelength; ↑, increase; ↓, decrease.

Ref.	Type	fNIRS		Study Design					Result	
		$N_{ch}$	λ [nm]	$N_{S}$	Age	Task	Region	Measurements	Physiological Alteration	Main Findings
[63]	Case Control	8	690, 830	14 CP, 14 TD	8–27 y	Lower extremity motor tasks	Bilateral SMC	HbO	↑ HbO amplitude in CP	SMC hyperactivation with muscle overflow
[64]	Case Control	42	690, 830	8 CP, 9 TD	12–25 y	Upper-limb motor tasks	Bilateral SMC	HbO	↑ HbO amplitude in CP	Marked SMC hyperactivation; lesioned hemisphere dominance
[65]	Case control	34	760, 850	8	5–15 y	RAGT	F, P, MC	HbO	Progressive ↑ activation across sessions	Training-induced cortical plasticity
[66]	Case control	-	750, 850	14 CP, 14 TD	7–11 y	LSUT	Bilateral PFC	HbO	↓ HbO amplitude in CP	Reduced PFC oxygenation during motor tasks
[67]	Case-control	34	760, 850	15 CP, 17 TD	3–10 y	Bilateral lower limb movement	L-PFC, R-PFC, L-MC, R-MC	FC	↓ FC and network efficiency	Compensatory R-MC reorganization
[68]	Case control	-	850, 760	14 CP, 14 TD	7–11 y	Robotic time-constrained reaching	L-PFC, R-PFC	HbO	↓ PFC activation under high time constraints	Ipsilateral PFC associated with better performance
[69]	Case control	27	760, 850	14 HCP, 28 TD	5–11 y	Upper limb mirror training	Bilateral PFC, MC	HbO, FC	PFC hypoactivation MC hyperactivation	Partial interhemispheric connectivity normalization

**Table 4:** Summary of HIE studies. HIE, hypoxic–ischemic encephalopathy; BG, basal ganglia; BP, blood pressure; CBV, cerebral blood volume; CPM, continuous physiological monitoring; CrSO_2_, cerebral tissue oxygenation; cSO_2_, cerebral oxygen saturation; EEG, electroencephalography; F, frontal; FPC, Fronto parietal cortex; FPN, frontoparietal network; FC, functional connectivity; FTOE, fractional tissue oxygen extraction; GA, gestational age; HbR, deoxygenated hemoglobin; HIBD, hypoxic–ischemic brain damage; HVx, hemoglobin volume index; HT, hypothermic therapy; L, left; L-PFC, left prefrontal cortex; LPL, left parietal lobe; LTL, left temporal lobe; MAP, mean arterial pressure; MAPOPT, optimal mean arterial pressure; MRI, magnetic resonance imaging; $N_{ch}$, number of channels; $N_{s}$, number of samples; oxCCO, oxidized cytochrome c oxidase; O, occipital; P, parietal; PPI, pressure passivity index; R, right; R-PFC, right prefrontal cortex; RPL, right parietal lobe; RrSO_2_, renal tissue oxygenation; rScO_2_, regional cerebral oxygen saturation; HbT, total hemoglobin; RTL, right temporal lobe; ScO_2_, cerebral hemoglobin oxygen saturation; SctO_2_, cerebral tissue oxygen saturation; T, temporal; TOI, tissue oxygenation index; w, weeks; λ, wavelength; ↑, increase; ↓, decrease; →, direction/flow.

Ref.	Type	fNIRS		Study Design					Result	
		$N_{ch}$	λ [nm]	$N_{S}$	Age	Task	Region	Measurements	Physiological Alteration	Main Findings
[70]	Retrospective	-	30	5 HT-treated, 6 non-HT	GA 35–41 w	Resting state	PC	ScO_2_, CBV	Early ↑ CBV/ScO_2_; partial recovery by 72 h	Early hyperemia indicated impaired autoregulation; hypothermia altered late perfusion
[71]	Prospective	-	-	36 HIE newborns	≥35 w GA	CPM	Bilateral Fronto-temporal cortex	HbR; autoregulation indices	↑ PPI/gain → impaired autoregulation	Impaired autoregulation linked to R-hemisphere vulnerability
[72]	Prospective	-	-	64 neonates	Term newborns	Continuous autoregulation monitoring	Bilateral Frontal cortex; MRI regions: paracentral gyri, white matter, BG, brainstem	AHVx, HbT, MAP	MAPOPT deviation ↑ injury; MAPOPT maintenance ↓ injury	Autoregulation-guided BP reduced MRI injury
[73]	Clinical	-	770–906	55 neonates	First 4 days of life	Resting state	R-Frontal hemisphere	TOI	TOI dependent on NIRS settings	Multi-distance NIRS improved reliability
[74]	Prospective	-	-	9 HIE neonates	≈39 w GA	Continuous monitoring	FPC	MAP-SctO_2_ coherence	Impaired autoregulation	Abnormal MAP-SctO_2_ coupling predicted brain injury
[75]	Prospective	-	Broadband	14 HIE infants	Term neonates	Continuous metabolic monitoring	Global cortical metabolism	Metabolic oxCCO + HbR coupling	Severe HIE: ↓ oxCCO-HbR coupling	↓ oxCCO-HbR coupling indicated mitochondrial dysfunction
[76]	Prospective	-	-	48 neonates	Term neonates	Resting state	Bilateral frontal Cortex	rScO_2_	↓ R-L rScO2 asymmetry	Destructive HIE lesions showed with reduced asymmetry
[77]	Retrospective	-	-	28 HIE neonates	GA ≥ 36 w	Resting state	F Cortex	rScO_2_	Progressive ↑ rScO_2_	Rising rScO_2_ during hypothermia predicted severe disability
[78]	Case control	45	760, 850	13 HIBD, 13 controls	GA 37–44 w	Resting state	L-PFC, R-PFC, LTL, RTL, LPL, RPL	Resting-state FC	↓ Global connectivity; focal hypercompensation	HIBD showed long-range disconnection
[79]	Prospective	-	-	58 HIE neonates	GA > 36 w	Resting state	R-frontal cortex	cSO_2_, FTOE	↑ cSO_2_ and ↓ FTOE (24 h)	Early oxygenation differences resolved by 18 h
[80]	Prospective	-	-	50 HIE neonates	Full term	Resting state	R-frontal area	CrSO^2^, RrSO_2_	Early CrSO_2_ changes; progressive ↑ RrSO_2_	Renal tissue oxygenation showed renal perfusion
[81]	Translational	-	-	Ovine: 5, Human: 14 neonates	GA > 35 w	Continuous multimodal monitoring	Bilateral FPN	Resting state neurovascular coupling	Severe HIE: ↓ neurovascular coupling	EEG-fNIRS differentiated HIE severity and detected hypoxia/seizures
[82]	Case–control	20	760, 850	21 HIE, 20 controls		Resting state	Bilateral F, P, T, O Cortex	Resting-state FC	↑ Short-range; ↓ long-range connectivity	fNIRS connectivity correlated with MRI/fMRI measures

**Table 5:** Summary of Ep studies. AG, anxiety group; CBV, cerebral blood volume; EEG, electroencephalography; FC, functional connectivity; FDR, false discovery rate; FLE, frontal lobe epilepsy; FTE, frontotemporal epilepsy; HbO, oxygenated hemoglobin; HbR, deoxygenated hemoglobin; IFG, inferior frontal gyrus; L, left; L-FTE, left frontotemporal epilepsy; m, months; MC, motor cortex; MLTE, mesial temporal lobe epilepsy; MTG, middle temporal gyrus; NAG, non-anxiety group; $N_{ch}$, number of channels; $N_{s}$, number of samples; PFC, prefrontal cortex; R, right; R-FTE, right frontotemporal epilepsy; RFE, refractory focal epilepsy; ROI, region of interest; RSFC, resting-state functional connectivity; SC, sensory cortex; STG, superior temporal gyrus; TC, temporal cortex; TD, typically developing; TLE, temporal lobe epilepsy; VFT, verbal fluency test; y, years; λ, wavelength; ↑, increase; ↓, decrease.

Ref.	Type	fNIRS		Study Design					Result	
		$N_{ch}$	λ [nm]	$N_{S}$	Age	Task	Region	Measurements	Physiological Alteration	Main Findings
[83]	Observational	44	695, 830	6 MLTE	21–55 y	Resting state with ictal monitoring	Bilateral TC	Ictal HbO and hemispheric ROI analysis	↑ HbO in epileptogenic hemisphere	↑ Ictal HbO enabled accurate seizure-focus lateralization
[84]	Intraoperative	N/A	N/A	3	6–28 y	Intraoperative Broca area stimulation with hemodynamic mapping	IFG, STG, MTG	HbO, HbR	↑ HbO ↓ HbR along pathway	Broca stimulation showed language-network reorganization
[85]	Prospective	N/A	N/A	6	1.5–8 m	Resting state	F cortex	HbO, CBV	Early ↑ HbO/Hb Altered neurovascular coupling	Early ↑ CBV followed by altered neurovascular coupling in infantile spasms
[86]	Prospective	N/A	690, 830	40 RFE	11–62 y	Seizure detection	Scalp-level cortical coverage	HbO, HbR	N/A	EEG-fNIRS improved seizure detection
[87]	Case control	52	695, 830	11 L-FTE, 11 R-FTE, 22 controls	N/A	VFT	Bilateral fronto-temporal language networks	FC	Lateralization-dependent reconfiguration	FTE showed hemisphere-dependent language reorganization
[12]	Case control	50	760, 850	20 FLE/TLE, 20 TD	6–18 y	Resting state and receptive language task	whole-brain connectivity	HbO based FC	↓ Interhemispheric/L connectivity ↑ R connectivity	↓ Interhemispheric connectivity with rightward network reorganization
[13]	Prospective	55	730, 850	18 AG, 20 NAG	10–60 y	Resting state	Bilateral PFC, MC, SC	HbO based FC	Subtle ↓ RSFC No FDR significance	Resting-state fNIRS showed limited anxiety-related connectivity changes

## Data Availability

The raw data supporting the conclusions of this article will be made available by the authors without undue reservation.

## Supplementary Materials

The following supporting information can be downloaded at: https://www.mdpi.com/article/10.3390/bios16080408/s1, Note S1: Detailed search criteria; Note S2: Inclusion and exclusion criteria; Note S3: Overview of Functional Near-Infrared Spectroscopy (fNIRS) Instrumentation; Note S4: Additional Context on Representative fNIRS Findings; Table S1: Cortical Regions Examined in fNIRS Studies (2015–2025). PRISMA 2020 Checklist: Identify the report as a systematic review.

## Review Registration

The study adhered strictly to PRISMA guidelines. To ensure full methodological transparency, the review protocol was registered retrospectively on the Open Science Framework (OSF; https://doi.org/10.17605/OSF.IO/J92BR).
