# Supplementary material for: Cortical Region Reporting Patterns in Neurodevelopmental Disorders: A Systematic Review of fNIRS Studies
Source: Biosensors (Basel). 2026 Jul 27;16(8):408. doi: 10.3390/bios16080408 (PMC13511062; doi:10.3390/bios16080408)
Supplement: Supplementary file 1 [file biosensors-16-00408-s001.zip › biosensors-4381136-supplementary.pdf]

[Supplementary Information]

Cortical Region Reporting Patterns in Neurodevelopmental Disorders: A Systematic Review of fNIRS Studies

Umm E Habiba[1], Nida Mateen[1], Keum-Shik Hong[2,3], Chang-Seok Kim[1,5], Jing Meng[4], Hwidon Lee[1,5,*], Jeesu Kim[1,5,*]

1 Department of Cogno-Mechatronics Engineering, Pusan National University, Busan 46241, Republic of Korea.
2 Institute for Industrial Artificial Intelligence, School of Mechanical Engineering, Pusan National University, Busan 46241, Republic of Korea.
3 Institute for Future, School of Automation, Qingdao University, Qingdao 266071, China.
4 School of Computer Science, Qufu Normal University, Rizhao 276826, China.
5 Engineering Research Center for Color-Modulated Extra-Sensory Perception Technology and Crystal Bank Research Institute, Pusan National University, Busan 46241, Republic of Korea.
* Correspondence: jeesukim@pusan.ac.kr (J.K.); hwidonlee@pusan.ac.kr (H.L.)

Note S1. Detailed Search Criteria.

Search terms for different databases (n=5).

PubMed

(((((((((((((((((Near-Infrared Spectroscopy) OR (NIRS)) OR (NIR)) OR (near infrared spectroscopy)) AND (Longitudinal Studies)) OR (longitudinal)) OR (trajectories)) OR (developmental)) OR (neurodevelopmental) AND ((y_10[Filter]) AND (ffrft[Filter]) AND (excludepreprints[Filter]) AND (fha[Filter]) AND (casereports[Filter] OR clinicalstudy[Filter] OR clinicaltrial[Filter]) AND (humans[Filter]) AND (english[Filter]))) OR (Autism Spectrum Disorder)) OR (Attention Deficit Disorder with Hyperactivity)) OR (Cerebral Palsy)) OR (Hypoxia-Ischemia)) OR (Epilepsy) AND ((excludepreprints[Filter] OR medline[Filter]) AND (casereports[Filter] OR clinicalstudy[Filter] OR clinicaltrial[Filter] OR observationalstudy[Filter] OR veterinaryobservationalstudy[Filter] OR randomizedcontrolledtrial[Filter]) AND (fft[Filter]) AND (humans[Filter]) AND (2015/1/1:2025/12/25[pdat]) AND (data[Filter]) AND (english[Filter]))) AND (neuroimaging)) AND (bioimaging)) OR (Optical imaging)) OR (fNIRS) Filters: Full text, Associated data, Case Reports, Clinical Study, Clinical Trial, Observational Study, Observational Study, Randomized Controlled Trial, English, Humans, Exclude preprints, MEDLINE, from 2015/1/1 - 2025/12/25 Sort by: Publication Date

PsycINFO/Psyc Articles

(NIRS OR NIR OR "near infrared spectroscopy") AND (longitudinal OR longitudinally OR "age groups" OR trajectory OR trajectories OR TI(developmental) OR TI(development) OR neurodevelopmental OR "developmental trajectory") AND (autism OR ASD OR "autism spectrum disorder" OR ADHD OR "attention-deficit/hyperactivity disorder" OR "cerebral palsy" OR CP OR "hypoxic-ischemic encephalopathy" OR HIE OR epilepsy) AND (PE >= 2015 AND PE <= 2025)

Scopus

TITLE-ABS (NIRS OR NIR OR "near infrared spectroscopy") AND TITLE-ABS (longitudinal OR longitudinally OR "age groups" OR trajectory OR trajectories OR developmental OR development OR neurodevelopmental OR "developmental trajectory") AND TITLE-ABS (autism OR ASD OR "autism spectrum disorder" OR ADHD OR "attention deficit hyperactivity disorder" OR "cerebral palsy" OR CP OR "hypoxic ischemic encephalopathy" OR HIE OR epilepsy AND PUBYEAR > 2015 AND PUBYEAR < 2025

Web of Science

(TI=(NIRS) OR TI=(NIR) OR TI=("near infrared spectroscopy")) AND (TI=(longitudinal) OR TI=(longitudinally) OR TI=("age groups") OR TI=(trajectory) OR TI=(trajectories) OR TI=(developmental) OR TI=(development) OR TI=(neurodevelopmental) OR TI=("developmental trajectory")) AND (TI=(autism) OR TI=(ASD) OR TI=("autism spectrum disorder") OR TI=(ADHD) OR TI=("attention deficit hyperactivity disorder") OR TI=("cerebral palsy") OR TI=(CP)OR TI=("hypoxic ischemic encephalopathy") OR TI=(HIE)OR TI=(epilepsy))ANDPY=2015-2025

Embase

('near infrared spectroscopy'/exp OR NIRS:ab,ti OR NIR:ab,ti OR "near infrared spectroscopy":ab,ti) AND ('longitudinal study'/exp OR longitudinal:ab,ti OR longitudinally:ab,ti OR 'age group':ab,ti OR trajectory:ab,ti OR trajectories:ab,ti OR developmental:ab,ti OR development:ti OR neurodevelopmental:ab,ti OR "developmental trajectory":ab,ti) AND ('autism spectrum disorder'/exp OR autism:ab,ti OR ASD:ab,ti OR 'attention deficit hyperactivity disorder'/exp OR ADHD:ab,ti OR 'cerebral palsy'/exp OR "cerebral palsy":ab,ti OR CP:ab,ti OR 'hypoxic ischemic encephalopathy'/exp OR HIE:ab,ti OR 'epilepsy'/exp OR epilepsy:ab,ti) AND [2015-2025]/py

Note S2: Inclusion and exclusion criteria

Inclusion Criteria

1. Publication Date
   - Published between January 2015 and December 2025
2. Population
   - Studies involving human participants diagnosed with:
     - Autism Spectrum Disorder (ASD)
     - Attention-Deficit/Hyperactivity Disorder (ADHD)
     - Cerebral Palsy (CP)
     - Hypoxic-Ischemic Encephalopathy (HIE)
     - Epilepsy (Ep)
   - It can include typically developing controls only if used as a comparison group.
3. Age Group
   - Focused on infants, children, and adolescents
   - Participants aged 0-18 years at the time of assessment
     - Studies including adults (>18 years) will be included only if: - Pediatric-onset condition (e.g., childhood epilepsy followed into adulthood)
4. Modality
   - Must use functional near-infrared spectroscopy (fNIRS) as the primary neuroimaging tool.
   - Multimodal studies (e.g., fNIRS + EEG) can be included if fNIRS results are reported separately.
5. Study Type
   - Empirical studies only (i.e., original research articles, trials, cohort studies).
   - Exclude reviews, editorials, opinion pieces, conference abstracts unless they report data.
6. Task Paradigm
   - Both resting-state and task-based paradigms (e.g., social, motor, cognitive tasks) are allowed.
   - Task Categories for Extraction:
     - Social cognition (face processing, joint attention, imitation)
     - Executive function (working memory, inhibition, cognitive control)
     - Motor (action execution, observation, motor learning)
     - Language (verbal fluency, comprehension, naming)
     - Sensory processing (visual, auditory)
     - Resting state (spontaneous fluctuations)
7. Outcomes
   - Must report at least one of the following:
     - Cortical activation (e.g., ΔHbO, ΔHbR)
       - Studies MUST report HbO (oxygenated hemoglobin) data
       - Studies reporting ONLY HbR or HbT
       - Task-based activation (HbO change during task vs. baseline/rest)
       - Resting-state
       - HbO fluctuations
       - Intervention effects on HbO
     - Must report at least one of the following:
       - Cortical activation (e.g., ΔHbO, ΔHbR) with anatomical localization
       - Minimum requirement: Broad region (frontal, temporal, parietal, motor, occipital)
     - Functional connectivity
8. Language
   - Published in English (or with English full-text available).

Exclusion Criteria

- Animal studies
- Purely methodological papers
- Studies with unclear clinical diagnosis or mixed populations without subgroup analysis
- fNIRS used only for validation purposes (e.g., signal quality testing)

Note S3: Overview of Functional Near-Infrared Spectroscopy (fNIRS) Instrumentation

Principle of fNIRS

Functional near-infrared spectroscopy (fNIRS) is a non-invasive optical neuroimaging technique that measures cerebral hemodynamic responses associated with neuronal activation through neurovascular coupling. By exploiting the differential absorption of near-infrared light (approximately 650–950 nm) by oxygenated (HbO) and deoxygenated hemoglobin (HbR), fNIRS provides an indirect measure of cortical activity using the modified Beer–Lambert law [8,10,95]. Owing to its portability, safety, relatively low cost, and tolerance to motion, fNIRS has become one of the most widely adopted functional neuroimaging modalities for pediatric research, enabling brain function assessment during naturalistic cognitive and behavioral paradigms that may be difficult to perform using conventional modalities such as functional magnetic resonance imaging (fMRI) [91,92]. Since all studies included in this systematic review employed fNIRS, understanding the underlying instrumentation is important for interpreting variations in cortical region reporting. In particular, differences in hardware configuration, probe geometry, signal acquisition, and data processing can influence cortical sensitivity, spatial localization, and the reproducibility of reported activation patterns [93,94,96].

Instrumentation Components and Optical Signal Acquisition

An fNIRS system primarily consists of light sources, photodetectors, optodes, and a data acquisition unit, all of which collectively determine measurement quality and cortical sensitivity [92,96]. Commercial systems typically employ either laser diodes or light-emitting diodes (LEDs) operating at two or more wavelengths (commonly 690–850 nm or 760–850 nm) to differentiate HbO and HbR concentration changes [94–96]. The emitted light propagates through biological tissues before being detected by highly sensitive photodetectors, where changes in optical intensity are converted into relative hemoglobin concentration changes. Detector sensitivity, optical power, sampling frequency, and acquisition electronics influence the signal-to-noise ratio (SNR), temporal resolution, and overall measurement reliability [96]. Recent advances in miniaturization and wireless connectivity have further expanded the applicability of fNIRS, with fully wearable systems now available for ambulatory and real-world monitoring [94,97]. Commercially available systems used in the included studies, including Hitachi, NIRx, Shimadzu, Artinis, and TechEn, vary in channel number, wavelength combinations, detector technology, sampling frequency, and acquisition software, highlighting the importance of considering instrumentation when interpreting regional activation patterns [91,93,96].

Probe Geometry and Cortical Coverage

Probe geometry is a key determinant of spatial localization and cortical sampling in fNIRS. Source–detector separation, optode density, channel number, and probe placement collectively influence photon penetration depth, cortical coverage, and spatial resolution [95,96]. A source–detector separation of approximately 30 mm is commonly adopted because it provides an optimal balance between cortical sensitivity and signal attenuation, while limiting measurements primarily to superficial cortical regions (approximately 1–2 cm beneath the scalp) [8,10,92]. Probe placement is generally guided by the international 10–20 EEG coordinate system to improve anatomical correspondence across participants, although variations in head size and cortical anatomy, particularly in pediatric populations, may still affect localization accuracy [10,93]. In addition, recent systems increasingly incorporate short-separation channels (typically 8–15 mm) to reduce contamination from superficial physiological signals and improve the specificity of cortical measurements [93]. These factors should be considered when comparing regional activation patterns across studies, as differences in probe geometry and cortical coverage may partly explain the predominance of prefrontal cortex investigations [91,93] observed in this systematic review.

System Architectures and Methodological Heterogeneity

Although all included studies utilized fNIRS to assess cortical function, considerable heterogeneity was observed in instrumentation and acquisition protocols. Three principal fNIRS system architectures are currently available: continuous-wave (CW), frequency-domain (FD), and time-domain (TD) systems [8,92,95]. CW-fNIRS, the most widely used platform in pediatric neuroimaging and across the studies included in this review, measures relative changes in light attenuation and provides estimates of HbO and HbR concentrations. Its portability, lower cost, and ease of operation have made it the preferred choice for developmental and clinical research [94,97]. In contrast, FD-fNIRS measures both light intensity and phase shift, enabling estimation of tissue optical properties, whereas TD-fNIRS records photon time-of-flight to provide improved depth sensitivity and more accurate quantification of optical parameters. Compared with CW systems, FD and TD systems offer improved quantification and depth sensitivity but require more sophisticated instrumentation, limiting their widespread adoption in pediatric clinical research [95,96].

Beyond system architecture, variability in wavelength selection, channel density, sampling frequency, probe layout, acquisition software, and manufacturer-specific hardware may further influence signal quality and spatial sampling [93–96]. Similarly, differences in preprocessing strategies including motion artifact correction, temporal filtering, baseline normalization, short-separation regression, and physiological noise removal can affect the reliability and reproducibility of hemodynamic measurements [10,91,93]. These methodological differences in both instrumentation and signal processing should be taken into account when comparing cortical activation findings across studies, as they may contribute to inter-study variability independent of the underlying neurodevelopmental disorder.

Technical Limitations and Future Perspectives

Despite its considerable advantages, fNIRS remains subject to several technical limitations. Because near-infrared light penetrates only superficial cortical tissue, fNIRS cannot directly assess deep brain structures such as the basal ganglia, hippocampus, and thalamus, limiting its application to the outer cerebral cortex [8,92,95]. Signal quality may also be affected by motion artifacts, systemic physiological fluctuations (e.g., cardiac pulsation, respiration, and scalp blood flow), anatomical variability, and differences in optode placement and preprocessing pipelines [91,93]. Recent advances in high-density fNIRS (HD-fNIRS), wearable wireless systems [94,97], improved short-separation regression, multimodal integration with EEG and MRI, and artificial intelligence-assisted signal analysis are expected to improve spatial localization, signal quality, and clinical applicability [92–96]. Continued standardization of instrumentation, acquisition protocols, and reporting practices as promoted by recent research and community initiatives [93] will further enhance the reproducibility of fNIRS studies and facilitate more reliable comparisons of cortical region reporting in future systematic reviews.

Note S4: Additional context on a few representative fNIRS studies' findings

Representative studies in ASD

Mori et al. [18] looked at young infants with ASD and usually developing controls. Through the application of fNIRS, the authors observed that during imitation, children with ASD had reduced oxyhemoglobin responses in the left inferior frontal gyrus (IFG). Following imitation training, there was an increase in IFG activation, which could be a sign of better recruitment of imitation-related networks and experience-dependent neuronal plasticity. Children with ASD showed reduced long-range temporal correlations in the temporal and temporo-occipital areas, according to Jia et al.'s [26] study of spontaneous brain activity during cartoon viewing. This raises the possibility of employing fNIRS-based temporal metrics as functional biomarkers and implies changes in the organization of brain activity with the passage of time. Wang et al. [29] investigated how parents and kids synchronize their brain activity during cooperative tasks using fNIRS hyperscanning. They discovered that the prefrontal cortical synchrony patterns of parents and children with ASD differed, and that lower synchrony was associated with more severe symptoms. This implies that fNIRS may aid in the understanding of social interaction-related brain systems.

Representative studies in ADHD

Using fNIRS, Yasumura et al. [54] examined prefrontal cortex activation in children with ADHD and normally developing controls during a Reverse Stroop test. Children with ADHD across age groups showed decreased activation in the right and medial prefrontal cortex, while higher activation in the left prefrontal cortex suggested potential compensatory recruitment. These results point to altered prefrontal executive control network development in ADHD and show how fNIRS may be used to describe age-related changes in cortical function. During a Go/No-go inhibitory control exercise, Cui et al. [59] looked at brain activity patterns in kids with ADHD. The authors found extensive decreases in task-evoked HbO responses including prefrontal and sensorimotor-related areas using fNIRS. Impaired recruitment of executive control networks during inhibitory processing is suggested by the observed prefrontal hypoactivation and modified functional connectivity patterns. Lee et al. [60] used fNIRS to examine prefrontal cortical responses in children with ADHD during a continuous performance test (CPT). In comparison to typically developing controls, children with ADHD showed decreased activation in the right dorsolateral prefrontal cortex (rDLPFC), according to the study. The disturbed coupling between frontal activation and attentional stability was linked to the decreased prefrontal response, indicating that prefrontal dysfunction is involved in attention regulatory deficiencies.

Representative studies in CP

Cortical remodeling linked to poor motor function has been the main focus of fNIRS investigations in children and young people with cerebral palsy (CP). Using fNIRS to study upper-limb motor activities, De Campos et al. [64] showed that individuals with CP had higher bilateral sensorimotor cortex oxyhemoglobin (HbO) activation than normally developing controls. The lesioned hemisphere mostly affected activation patterns, indicating adaptive recruitment of remaining motor networks after brain injury. Zhang et al. [69] revealed changed activation patterns involving both prefrontal and motor cortices after further examining cortical responses during upper-limb mirror training tasks. During mirror tasks, CP participants displayed decreased prefrontal activation, although compensatory hyperactivation was seen in motor areas along with altered interhemispheric connectivity.

Representative studies in HIE

fNIRS has been used mainly for ongoing bedside monitoring of cerebral oxygenation, autoregulation, and metabolic function in critically unwell neonates with hypoxic–ischemic encephalopathy (HIE). Tian et al. [74] assessed mean arterial pressure and cerebral oxygenation coupling using continuous fNIRS monitoring during therapeutic hypothermia. Wavelet-based analysis revealed abnormal autoregulatory patterns that were linked to later brain damage, indicating the possibility of fNIRS-derived hemodynamic parameters for early risk assessment. Mitra et al. [75] used broadband near-infrared spectroscopy to further examine metabolic changes in infants with HIE following therapeutic hypothermia. Infants with more severe HIE showed reduced coupling between hemoglobin-derived signals and oxidized cytochrome c oxidase (oxCCO), indicating chronic mitochondrial dysfunction and bolstering the potential of metabolic fNIRS measurements as indicators of damage severity.

Representative studies in Ep

Particularly in early-life epileptic diseases, fNIRS has been investigated as an additional technique for assessing altered cortical hemodynamics and neurovascular function in epilepsy. Using resting-state fNIRS, Ponchel et al. [85] examined babies with epileptic spasms and found two different hemodynamic phases that were distinguished by early increases in cerebral blood volume and subsequent changes in neurovascular coupling. These results demonstrated the feasibility of combined EEG–fNIRS monitoring for evaluating early epileptic brain dysfunction and revealed diffuse cortical involvement. Using resting-state and language-related fNIRS paradigms to measure functional connectivity within language networks, Hüsser et al. [12] examined children with frontal or temporal lobe epilepsy. A rightward shift in language network organization was shown by the study, which showed decreased interhemispheric and left-hemisphere connection along with higher right-hemisphere network efficiency. These results imply that compensatory cortical rearrangement linked to epilepsy can be captured by fNIRS, which may offer insights into cognitive outcomes and functional adaptation.

Table S1: Cortical Regions Examined in fNIRS Studies (2015–2025). ASD, autism spectrum disorder; ADHD, attention-deficit/hyperactivity disorder; CP, cerebral palsy; HIE, hypoxic-ischemic encephalopathy; Ep, epilepsy; ☑, region examined in at least one study; ☐, not reported; n, number of disorders reporting the region

Lobe	Region	ASD	ADHD	CP	HIE	Ep	n
**Frontal**	Prefrontal Cortex	☑	☑	☑	☑	☑	5
	Inferior Frontal Gyrus	☑	☑	☐	☐	☑	3
	Precentral Gyrus (Motor)	☐	☐	☑	☑	☐	2
	Dorsolateral PFC	☑	☑	☐	☐	☐	2
	Frontopolar Cortex	☑	☑	☐	☐	☐	2
	Medial PFC	☑	☑	☐	☐	☐	2
	Middle Frontal Gyrus	☑	☑	☐	☐	☐	2
	Sensorimotor Cortex	☐	☐	☑	☐	☑	2
	Premotor Cortex	☑	☐	☐	☐	☐	1
	Frontoparietal Cortex	☐	☐	☐	☑	☐	1
	Superior Frontal Gyrus	☐	☑	☐	☐	☐	1
	Temporoparietal Junction	☑	☐	☐	☐	☐	1
	Ventrolateral PFC	☐	☑	☐	☐	☐	1
**Parietal**	Parietal Cortex	☑	☑	☑	☑	☐	4
	Postcentral Gyrus (Sensory)	☑	☐	☐	☐	☑	2
	Supramarginal Gyrus	☑	☑	☐	☐	☐	2
	Angular Gyrus	☐	☑	☐	☐	☐	1
	Inferior Parietal Lobule	☑	☐	☐	☐	☐	1
**Temporal**	Temporal Cortex	☑	☑	☐	☑	☑	4
	Middle Temporal Gyrus	☑	☐	☐	☐	☑	2
	Superior Temporal Gyrus	☑	☐	☐	☐	☑	2
	Superior Temporal Sulcus	☑	☐	☐	☐	☐	1
**Occipital**	Occipital Cortex	☑	☐	☐	☑	☐	2
	Visual Association Cortex	☑	☐	☐	☐	☐	1
